# The best platinum regimens for chemo-naive incurable non-small cell lung cancer: network meta-analysis

**DOI:** 10.1038/s41598-017-13724-2

**Published:** 2017-10-13

**Authors:** Nobuyuki Horita, Akimichi Nagashima, Kentaro Nakashima, Yuji Shibata, Kentaro Ito, Atsushi Goto, Takeharu Yamanaka, Takeshi Kaneko

**Affiliations:** 1 Department of Pulmonology, Yokohama City University Graduate School of Medicine, Yokohama, Japan; 2 Respiratory Center, Matsusaka Municipal Hospital, Mie, Japan; 3 Division of Epidemiology, Center for Public Health Sciences, National Cancer Center, Tokyo, Japan; 4 Department of Biostatistics, Yokohama City University, Yokohama, Japan

## Abstract

Platinum regimens still play a key role in chemotherapy for incurable non-small cell lung cancer (NSCLC). Although guidelines list many platina regimens, the best regimens have not yet clarified. Electronic searches were carried out during November 26th-28th, 2016. We included individually randomized trials comparing two or more platinum regimes for incurable chemo-naive NSCLC published in English full papers. The platinum doublets should be either Cisplatin (CDDP), Carboplatin (CBDCA), or Nedaplatin (CDGP) plus one of the third-generation agents. The platinum triplet should be the doublet plus bevacizumab (BEV). The data were independently extracted and cross-checked by two investigators. We did not observed heterogeneity (whole network level Q = 28.9, df = 34, P = 0.717) among 59 pairwise comparisons from 45 studies with 16141 cases for the primary outcome, hazard ratio for overall survival (HRos). Using CBDCA + Paclitaxel (PTX) + BEV as a common comparator, CDGP + Docetaxel (DTX) (HRos = 0.98, 95%CI: 0.75–1.29, P = 0.884), CDDP + Tegafur gimeracil oteracil (S1) (HRos = 1.23, 95%CI: 0.96–1.57, P = 0.099), CBDCA + S1 (HRos = 1.23, 95%CI: 0.99–1.53, P = 0.062), and CDGP + Gemcitabine (GEM) (HRos = 1.24, 95%CI: 0.71–2.17, P = 0.45) did not have significantly poorer HRos. We suggest that these regimens as acceptable first-choice regimens.

## Introduction

Non-small cell lung cancer (NSCLC), which is currently the most common malignant neoplasm in the world, is one of the leading causes of cancer death worldwide^1^. In more than a half of cases, the NSCLC is detected after the disease has already progressed to an incurable stage. For such patients, chemotherapy is usually the first-choice treatment option because accumulated evidence has revealed that current standard chemotherapy treatments have substantial benefits for advanced, locally advanced, and recurrent NSCLC. Traditionally, some of the platinum regimens have been regarded as the standard first-line regimens for NSCLC for non-elderly patients with good performance status who do not have major co-morbidities. The currently preferred platinum doublets are combinations of one of the platinum agents and one of the third-generation chemotherapy agents. Currently accepted platinum triplets are combinations of these platinum doublets and bevacizumab (BEV)^2,3^. Even though epidermal growth factor receptor-tyrosine kinase inhibitors, anaplastic lymphoma kinase inhibitors, and immune check-point inhibitors have recently been preferred for certain subgroups of NSCLC patients, these platinum regimens still play a key role in chemotherapy for incurable NSCLC^2,3^.

Current guidelines list many platinum doublets and triplets as recommended therapeutic options for advanced NSCLC. However, the single best regimen among platinum regimens has not yet been clarified for various reasons: inconsist results from trials, difficulty in interpreting results from non-inferiority trials, lack of statistical power to detect subtle survival difference, differences of inclusion criteria such as performance status and age, and inconsistency of primary outcomes of original trials. To solve this, meta-analysis is a useful method to identify the best regimen^4^. Nonetheless, classical head-to-head pairwise meta-analysis cannot satisfactorily answer this clinical question because of the deficiency of direct comparisons among the numerous potentially best regimens. Therefore, some previous meta-analyses compared groups of regimens, for example, cisplatin (CDDP) regimens versus carboplatin (CBDCA) regimens or BEV regimens versus non-BEV regimens^5,6^. On the other hand, network meta-analysis is a recently developed technique to integrate available data. This analysis has the advantages of allowing collective comparison among multiple treatment arms and the potential precision gains from combining direct and indirect evidence^7–9^.

The goal of the current network meta-analysis is to identify and rank the best standard regimens by comparing the effectiveness and safety of a variety of the platinum regimens as first-line chemotherapies for advanced, locally advanced, and recurrent NSCLC.

## Material and Methods

### Protocol registration

This protocol of the systematic review and network meta-analysis has been uploaded on the website of International Prospective Register of Systematic Reviews (42016052455)^10^. We have composited this protocol following the Preferred Reporting Items for Systematic Reviews and Meta-Analyses statement and that for network meta-analysis^11,12^. Institutional Review Board approval and patient informed consent were waived due to the review nature of this study.

### Study search

Search formulas for electronic databases were created with the support of Cochrane Japanese. Search formulas for MEDLINE, EMBASE, the Web of Science Core Collection, and the Cochrane Central Register of Controlled Trials are presented in Supplementary Text 1. The search for each electronic database was carried out during November 26th-28th, 2016.

An additional manual search was conducted by two investigators (NH and AN) independently.

Candidate articles were first screened and then scrutinized independently by the two investigators. Discrepancies found during the study selection process were resolved by discussion between the two investigators.

### Inclusion criteria

#### Publication type and trial design

We included individually randomized trials comparing two or more platinum regimes for incurable NSCLC, which have been reported and published in English full papers. We allowed a trial with three or more arms. We allowed all of superiority, non-inferiority, phase II, phase III, non-blinded, single-blinded, and double-blinded trials. A trial evaluating only the co-secondary outcomes of our analysis was allowed. Included patients should have been randomized before the first-line chemotherapy initiation. Thus, randomization just before the maintenance therapy was not accepted. We carefully checked for and avoided duplicate uses of the same study.

#### Treatments

Our concern was with the first-line platinum doublet and triplet chemotherapy regimens. Platinum agents should be either CDDP, CBDCA or Nedaplatin (CDGP)^5,13^. We disregarded regimens with Oxaliplatin. The counterpart of the platinum doublet had to be one of the following third-generation chemotherapy agents: Docetaxel (DTX), Paclitaxel (PTX), Vinorelbine (VNR), Gemcitabine (GEM), Irinotecan (CPT-11), Pemetrexed (PEM), and Tegafur gimeracil oteracil (S1)^2,3^. We regarded albumin-bound PTX and PTX-poliglumex as PTX. A platinum triplet had to be a combination of a platinum doublet and BEV^2,3^. We did not include the following regimens: single agent chemotherapies, non-platinum doublets/triplets, regimens without the third-generation chemotherapy agent, regimens with Oxaliplatin, regimens with immune check-point inhibitors, and regimens that contained any targeted therapies for NSCLC with oncogenic driver mutation such as tyrosine kinase inhibitors. Any perioperative chemotherapy, adjuvant chemotherapy, neo-adjuvant chemotherapy, and radio-chemotherapy were also excluded. We did not include studies that planned to stop the first-line regimen before administration of the third course.

Regimens that used the same medication were evaluated collectively regardless of administration root, speed, dosage, and schedule. We classified the treatment regimen based on the first-line chemotherapy regardless of maintenance, second-line, and later-line treatment. Similarly, we focused only on the first-line regimen of the cross-over trial. We equated placebo with “no treatment.” For example, “CDDP + PEM + (placebo of BEV)” arm was identical to the “CDDP + PEM” arm for our analysis.

#### Patients

Chemo-naive patients with advanced, or locally-advanced, or recurrent NSCLC were included. Although the tumor, node, metastasis classification has been updated every 4–8 years, we accepted the TNM classification regardless of version difference. Recurrent cancer patients with a history of operation or radiotherapy were accepted unless these patients had never undergone chemotherapy. Patients with a history of any adjuvant chemotherapy, neoadjuvant chemotherapy, or radio-chemotherapy were excluded. The age, sex, performance status, co-morbidities, and organ functions of patients were not questioned. Any study focusing on patients with large cell neuroendocrine carcinoma was planed to be excluded though this carcinoma is usually classified as NSCLC. If a regimen included PEM or BEV, the pathological type had to be limited to adenocarcinoma or non-squamous carcinoma^14,15^.

### Quality assessment

We assessed the quality of original studies using six domains of the Cochrane Risk of Bias evaluation sheet: selection, performance, detection, attrition, reporting, and other biases^4^.

### Outcomes

The primary outcome was hazard ratio (HR) for overall survival (OS, HR_OS_)^16^.

The co-secondary outcomes were HR for progression-free survival (FPS, HR_PFS_)^16^, odds ratio (OR) for response rate (RR, OR_RR_)^17^, and OR for severe adverse event (SAE) including neutropenia, anemia, thrombopenia, febrile neutropenia, and nausea. Adverse events with a severity, defined with Common Terminology Criteria for Adverse Events, of grade-three or higher were counted^18^.

Evaluation of disease progression to assess the PFS and evaluation of objective response to assess RR should not have greatly deviated from the Response Evaluation Criteria In the Solid Tumors 2000 guidelines and the 2009 revised guidelines^17^. Time to progression and time to treatment failure were not regarded as PFS. When disease progression and objective response were evaluated both by physicians caring for the patients and by the blinded independent central review board, we chose the data based on a pre-specified endpoint in each original report. If this was not clear, we used data from the blinded independent central review. The number of SAEs were counted on a patient basis, not on a per-cycle basis.

### Data extraction

Data for the included studies, such as author name, publication year, country of origin, numbers of patients randomized, chemotherapy regimen, and data related to the study outcomes such as OS, PFS, RR, and SAE were extracted by the two investigators (NH and AN) independently. The data extracted by the two investigators were cross-checked and any discrepancies were discussed between them. We extracted data from non-inferiority studies using the same method as for superiority trials. For studies with three or more arms, data on every pairwise comparison were extracted. For example, a four-arm trial provided six comparisons. When only two arms of a three-arm study were of interest to us, we only used data of the two arms. For example, if a three-arm study evaluated CDDP + GEM, CDDP + DTX, and DTX monotherapy, we used only the data concerning CDDP + GEM and CDDP + DTX. When two arms used the same anti-cancer medication in a three-arm trial, the outcomes in the two arms sharing the same medication were merged prior to the main analysis. For example, for a three-arm trial with (a) CDDP + PEM + high-dose BEV, (b) CDDP + PEM + low-dose BEV, and (c) CDDP + GEM; (a + b) and (c) were compared. When updated data for survival was available, the most recently updated data were preferred. When necessary, we adopted Parmar’s method to obtain survival data^19^. Intention-to-treat analysis was preferred over full-analysis-set analysis and per-protocol analysis when two or more of these were available.

### Statistical analyses

We pooled the logarithm of OR, HR, and their SE using the frequentist weighted least squares approach random-model network meta-analysis^7–9^. All the binary outcomes were transformed to OR preceding the network meta-analysis. When one or more cells in a two-by-two contingency were zero, 0.5 was added to all the cells. When a network diagram showed two or more independent loops, we evaluated only the loop that contained major platinum regimes such as CDDP + PEM and CBDCA + PTX + BEV^2,3^. A league table for the primary outcome was presented with a 95%CI and P value. For a forest plot, a CDDP/CBDCA chemotherapy regimen with the best primary outcome performance was selected as the common reference comparator. A CDGP regimen could not be a common comparator, because the limited number of studies and patients evaluated for CDGP would make the confidence interval wider. The common reference comparator was also used for the forest plot for secondary outcomes. For the network meta-analysis, the “netmeta” command in the “netmeta” package of R was used^20^ (Supplementary Text 2).

Sensitivity analyses are planned: (i) Fixed-model network meta-analysis instead of random-model. (ii) Random-model network meta-analysis using data from the phase III trials.

## Results

### Study search

**Figure 1:**
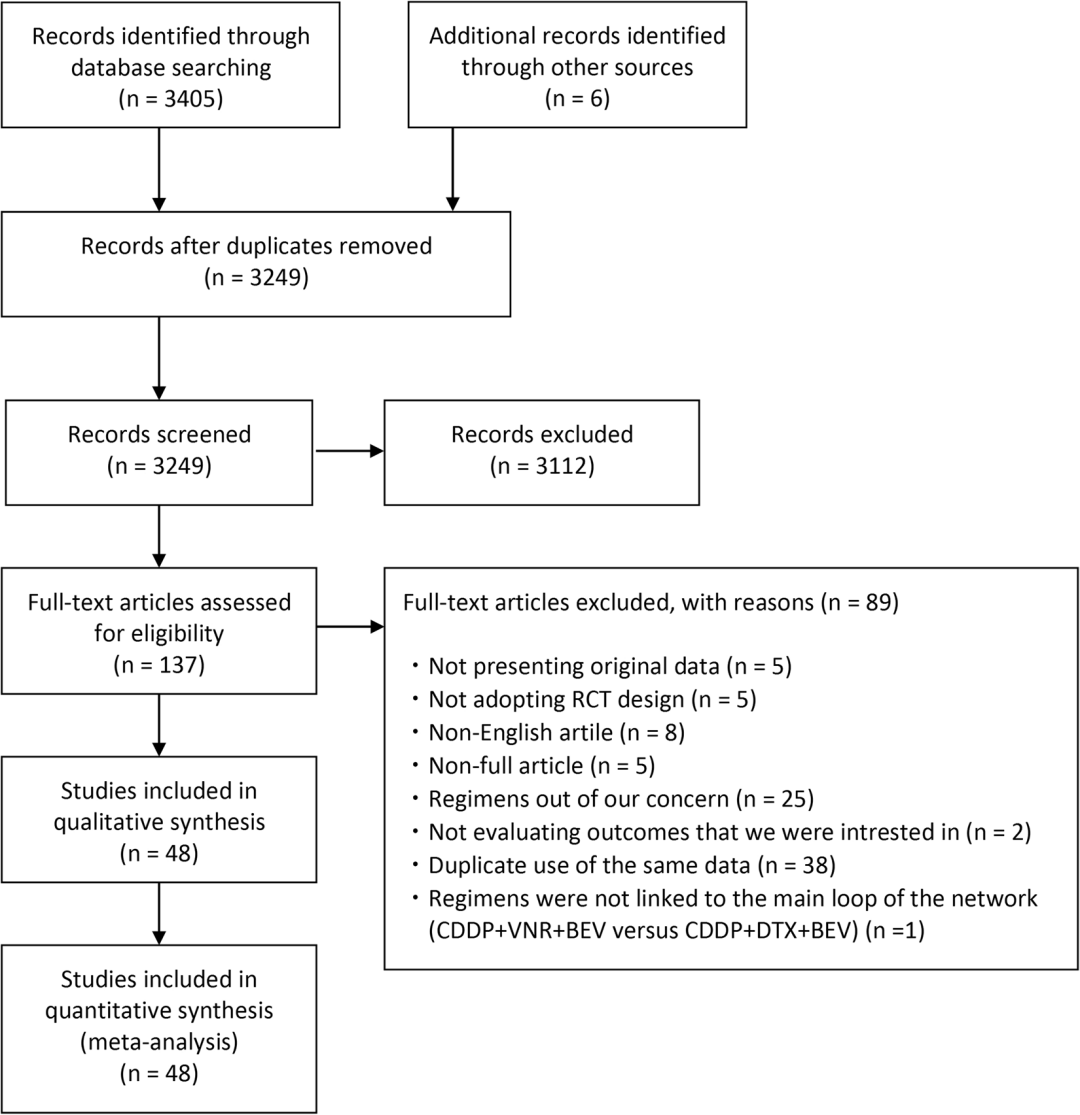
Preferred Reporting Items for Systematic Reviews and Meta- Analyses flow chart for study search.

We first found 3405 and six articles by electronic and hand searches, respectively. Of 3411 articles that met the preliminary criteria, 162, 3112, and 89 were excluded through removal of duplication, title/abstract screening, and full-article scrutinizing, respectively (Fig. 1). We finally found 48 eligible articles (Fig. 1, Table 1, Supplementary Text 3).

**Table 1:** Characteristics of included studies.

Study	Country	Phase	Primary outcome	Pathology	Arm	Stage,	Performance status	Regimens	Patients	median age
Bennouna (2014) NAVorial 01	France	II	DCR	NSq	2	IIIb, IV, Rec	KPS ≥ 80%	CDDP (75 mg/m2), PEM (500 mg/m2)	153	62
								CDDP (80 mg/m2), VNR (80 mg/m2 (d 1,8 po))		
Biesma (2011) NVALT-3	Netherlands	III	QOL	NSCLC	2	III, IV	ECOG 0-2	CBDCA (AUC 5), GEM (1250 mg/m2 (d 1,8))	182	74
								CBDCA (AUC 5), PTX (175 mg/m2)		
Chang (2008)	China	NS	RR	NSCLC	2	IIIb, IV	ECOG 0-2	CDDP (80 mg/m2), GEM (1000 mg/m2 (d 1,8,15))	83	62
								CDDP (80 mg/m2), VNR (20 mg/m2 (d 1,8,15))		
Chen (2004)	Taiwan	II	NS	NSCLC	2	IIIb, IV	ECOG 0-2	CDDP (60 mg/m2 (d15)), PTX (66 mg/m2 (d 1,8,15))	140	65
								CDDP (60 mg/m2 (d15)), VNR (23 mg/m2 (d 1,8,15))		
Chen (2006)	Taiwan	II	Neuropathy	NSCLC	2	IIIb, IV	ECOG 0-2	CBDCA (AUC 6), PTX (160 mg/m2)	81	75
								CDDP (60 mg/m2), PTX (160 mg/m2)		
Chen (2007)	Taiwan	II	RR	NSCLC	2	IIIb, IV	ECOG 0-2	CDDP (60 mg/m2), VNR (25 mg/m2 (d 1,8))	94	63
								CDDP (60 mg/m2), DTX (60 mg/m2)		
Comella (2000)	Italy	III	OS	NSCLC	3 ≫ 2 (excluded)	IIIb, IV	ECOG 0-1	CDDP (120 mg/m2), VNR (30 mg/m2 (weekly))	120	62
								CDDP (100 mg/m2), GEM (1000 mg/m2 (d 1,8,15))		
Douillard (2005)	France	II	RR	NSCLC	2	IV	ECOG 0-2	CDDP (100 mg/m2), DTX (75 mg/m2)	239	57
								CDDP (100 mg/m2), VNR (30 mg/m2 (d 1,8))		
Edelman (2004)	USA	II	OS	NSCLC	2	IIIb, IV	ECOG 0-1	CBDCA (AUC 5.5), GEM (1000 mg/m2(d 1,8))	204	60
								CDDP (100 mg/m2), VNR (25 mg/m2 (d 1,8))		
Fossella (2003) TAX 326	USA	III	OS (Non-inf)	NSCLC	3	IIIb, IV, Rec	KPS ≥ 70%	CDDP (75 mg/m2), DTX (75 mg/m2)	1218	60
								CBDCA (AUC 6), DTX (75 mg/m2)		
								CDDP (100 mg/m2), VNR (25 mg/m2 (weekly))		
Galetta (2015) ERACLE	Italy	III	QOL	NSq	2	IIIb, IV	ECOG 0-1	CDDP (75 mg/m2), PEM (500 mg/m2)	118	62
								CBDCA (AUC 6), PTX (200 mg/m2), Bev (15 mg/kg)		
Gebbia (2003)	Italy	III	TTP, OS	NSCLC	4 ≫ 2 (excluded)	IIIb, IV	ECOG 0-2	CDDP (100 mg/m2), VNR (25 mg/m2 (d 1,8))	278	62
								CDDP (100 mg/m2), GEM (1400 mg/m2 (d 1,8))		
Gebbia (2010)	Italy	II	QOL, AE, symptom	NSCLC	2	IIIb, IV	ECOG 0-1	CDDP (75 mg/m2), DTX (75 mg/m2)	86	62
								CDDP (80 mg/m2), VNR (30 mg/m2 (d 1,8))		
Gronberg (2009)	Norway	III	QOL,	NSq #	2	IIIb, IV	ECOG 0-2	CBDCA (AUC 5), PEM (500 mg/m2)	329	65
								CBDCA (AUC 5), GEM (1000 mg/m2 (d 1,8))		
Helbekkmo (2007)	Norwegian	III	OS	NSCLC	2	IIIb, IV	ECOG 0-2	CBDCA (AUC 5), VNR (25 mg/m2 (d 1,8))	444	67
								CBDCA (AUC 5), GEM (1000 mg/m2 (d 1,8))		
Johnson (2004) AVF-0757g	USA	II	TTP, RR	NSq #	3 ≫ 2 (combined)	IIIb, IV, Rec	ECOG 0-2	CBDCA (AUC 6), PTX (200 mg/m2), BEV (7.5 or 15 mg/kg)	79	63
								CBDCA (AUC 6), PTX (200 mg/m2)		
Kader (2013)	Egypt	II	Toxicity, PFS	NSq	2	IIIb, IV	ECOG 0-2	CBDCA (AUC 5), PTX (60 mg/m2), BEV (7.5 mg/kg)	41	52
								CDDP (75 mg/m2), PEM (500 mg/m2)		
Kawahara (2013)	Japan	II	PFS	NSCLC	2	IIIb, IV, Rec	ECOG 0-1	CBDCA (AUC 6), DTX (60 mg/m2)	90	67
								CBDCA (AUC 6), PTX (200 mg/m2)		
Khodadad (2014)	Iran	NS	PFS	NSCLC	2	IIIb, IV	ECOG 0-2	CDDP (75 mg/m2), DTX (75 mg/m2)	100	51
								CBDCA (AUC 5), PTX (200 mg/m2)		
Kubota (2015)CATS, TCOG0701	Japan	III	OS (Non-inf)	NSCLC	2	IIIb, IV, Rec	ECOG 0-1	CDDP (60 mg/m2 (d8)), S1 (80 mg/m2 (d 1-14 po bid))	608	62
								CDDP (80 mg/m2), DTX (60 mg/m2)		
Langer (2007) ECOG1599	USA	II	OS	NSCLC	2	IIIb, IV, Rec	ECOG 2	CBDCA (AUC 6), PTX (200 mg/m2)	103	66
								CDDP (60 mg/m2), GEM (1000 mg/m2 (d 1,8))		
Martoni (2005)	Italy	III	OR, TTP, ¶	NSCLC	2	IIIb, IV, Rec	KPS ≥ 70%	CDDP (75 mg/m2), VNR (25 mg/m2 (d 1,8))	286	63
								CDDP (75 mg/m2), GEM (1200 mg/m2 (d 1,8))		
Mazzanti (2003)	Italy	II	RR	NSCLC	2	IIIb, IV	ECOG 0-2	CDDP (80 mg/m2), GEM (1200 mg/m2 (d 1,8))	125	63
								CBDCA (AUC 5), GEM (1200 mg/m2 (d 1,8))		
Minami (2013)	Japan	II	PFS	NSCLC	2	IIIb, IV	ECOG 0-1	CBDCA (AUC 6), PTX (200 mg/m2)	50	64
								CBDCA (AUC 5), GEM (1000 mg/m2 (d 1,8))		
Niho (2012) JO19907	Japan	II	PFS	NSq	2	IIIb, IV, Rec	ECOG 0-1	CBDCA (AUC 6), PTX (200 mg/m2), BEV (15 mg/kg)	180	61
								CBDCA (AUC 6), PTX (200 mg/m2)		
Ohe (2007)FACS	Japan	III	OS (Non-inf)	NSCLC	4	IIIb, IV	ECOG 0-1	CDDP (80 mg/m2), CPT-11 (60 mg/m2 (d1,8,15))	602	62
								CBDCA (AUC 6), PTX (200 mg/m2)		
								CDDP (80 mg/m2), GEM (1000 mg/m2 (d 1,8))		
								CDDP (80 mg/m2), VNR (25 mg/m2 (d 1,8))		
Okamoto (2010) LETS 2013 Updated	Japan	III	OS (Non-inf)	NSCLC	2	IIIb, IV	ECOG 0-1	CBDCA (AUC 5), S1 (80 mg/m2 (d 1-14 po bid))	564	64
								CBDCA (AUC 6), PTX (200 mg/m2)		
Patel (2013) PointBreak	USA	III	OS	NSq	2	IIIb, IV	ECOG 0-1	CBDCA (AUC 6), PEM (500 mg/m2), BEV (15 mg/kg)	939	65
								CBDCA (AUC 6), PTX (200 mg/m2), BEV (15 mg/kg)		
Reck (2009)AVAiL	Germany	III	PFS	NSq	3 ≫ 2 (combined)	IIIb, IV, Rec	ECOG 0-1	CDDP (80 mg/m2), GEM (1250 mg/m2 (d 1,8)), BEV (7.5/15 mg/kg)	1043	58
								CDDP (80 mg/m2), GEM (1250 mg/m2 (d 1,8))		
Rodrigues (2011)	Argentina	III	G3/4PFS	NSq	2	IIIb, IV	ECOG 0-2	CBDCA (AUC 5), PEM (500 mg/m2)	260	60
								CBDCA (AUC 5), DTX (75 mg/m2)		
Rosell (2002)	Spain	III	RR (Non-inf)	NSCLC	2	IIIb, IV, Rec	ECOG 0-2	CDDP (80 mg/m2), PTX (200 mg/m2)	618	58
								CBDCA (AUC 6), PTX (200 mg/m2)		
Sandler (2010)E4599, Updated	USA	III	OS	Ad #	2	IIIb, IV	ECOG 0-1	CBDCA (AUC 6), PTX (200 mg/m2), BEV (15 mg/kg)	602	63
								CBDCA (AUC 6), PTX (200 mg/m2)		
Scagliotti (2002)	Italy	III	NS	NSCLC	3	IIIb, IV, Rec	ECOG 0-2	CDDP (75 mg/m2), GEM (1250 mg/m2 (d 1,8))	612	63
								CBDCA (AUC 6), PTX (225 mg/m2)		
								CDDP (100 mg/m2), VNR (25 mg/m2 (weekly))		
Scagliotti (2008)	Italy	III	OS (Non-inf)	NSq #	2	IIIb, IV	ECOG 0-1	CDDP (75 mg/m2), PEM (500 mg/m2)	1252	61
								CDDP (75 mg/m2), GEM (1250 mg/m2 (d 1,8))		
Schiller (2002) ECOG 1594	USA	NS	OS	NSCLC	4	IIIb, IV, Rec	ECOG 0-2	CDDP (75 mg/m2 (d 2)), PTX (135 mg/m2)	1207	63
								CDDP (75 mg/m2), GEM (1000 mg/m2 (d 1,8,15))		
								CDDP (75 mg/m2), DTX (75 mg/m2)		
								CBDCA (AUC 6), PTX (225 mg/m2)		
Schuette (2013)	Germany	II	PFS	NSq #	2	IIIb, IV	ECOG 0-1	CDDP (75 mg/m2), PEM (500 mg/m2)	133	64
								CBDCA (AUC 6), PEM (500 mg/m2)		
Shukuya (2015) WJCOG5208L	Japan	III	OS	Sq	2	IIIb, IV, Rec	ECOG 0-1	CDGP (100 mg/m2), DTX (60 mg/m2)	355	64
								CDDP (80 mg/m2), DTX (60 mg/m2)		
Smit (2003) EORTC08975	Netherlands	III	OS	NSCLC	3 ≫ 2 (excluded)	IIIb, IV	ECOG 0-2	CDDP (80 mg/m2), PTX (175 mg/m2)	319	57
								CDDP (80 mg/m2), GEM (1250 mg/m2 (d 1,8))		
Sun (2015)	Korea	II	RR	NSq	2	IIIb, IV, Rec	ECOG 0-1	CDDP (70 mg/m2), PEM (500 mg/m2)	321	60
								CDDP (70 mg/m2), GEM (1000 mg/m2 (d 1,8))		
Tan (2009)GLOB3	Singapore	III	TTF	NSCLC	2	IIIb, IV, Rec	KPS ≥ 80%	CDDP (80 mg/m2), VNR (30 (d1), 80 (d 8 po) mg/m2)	390	61
								CDDP (75 mg/m2), DTX (75 mg/m2)		
Thomas (2006) GFPC99-01	France	II	RR	NSCLC	2	IIIb, IV	ECOG 0-2	CBDCA (AUC 6), GEM (1250 mg/m2 (d 1,8))	100	58
								CDDP (80 mg/m2), VNR (30 mg/m2 (weekly))		
Treat (2010)	USA	III	OS	NSCLC	3 ≫ 2 (excluded)	IIIb, IV, Rec	ECOG 0-2	CBDCA (AUC 5.5), GEM (1000 mg/m2 (d 1,8))	758	64
								CBDCA (AUC 6), PTX (225 mg/m2)		
Wu (2014) JMIL	China	III	OS	NSq	2	IIIb, IV	ECOG 0-1	CDDP (75 mg/m2), PEM (500 mg/m2)	256	57
								CDDP (75 mg/m2), GEM (1250 mg/m2 (d 1,8))		
Yang (2012)	China	NS	RR	NSCLC	2	IIIb, IV	ECOG 0-2	CDGP (80 mg/m2), GEM (1250 mg/m2 (d 1,8))	62	57
								CBDCA (AUC 5), GEM (1250 mg/m2 (d 1,8))		
Zatloukal (2003)	Czech	III	G3/4 toxicity	NSCLC	2	IIIb, IV	KPS ≥ 70%	CDDP (80 mg/m2), GEM (1200 mg/m2 (d 1,8))	176	62
								CBDCA (AUC 5), GEM (1200 mg/m2 (d 1,8))		
Zhang (2013)	China	II	PFS	NSq #	2	IIIb, IV, Rec	ECOG 0-1	CDDP (75 mg/m2), PEM (500 mg/m2)	205	54
								CDDP (75 mg/m2), GEM (1000 mg/m2 (d 1,8))		
Zhou (2015)BEYOND	China	III	PFS	NSq	2	IV, Rec	ECOG 0-1	CBDCA (AUC 6), PTX (175 mg/m2), BEV (15 mg/kg)	276	57
								CBDCA (AUC 6), PTX (175 mg/m2)		
Zinner (2015) PRONOUNCE	USA	III	G4PFS	NSq	2	IV	ECOG 0-1	CBDCA (AUC 6), PTX (200 mg/m2), BEV (15 mg/kg)	361	66
								CBDCA (AUC 6), PEM (500 mg/m2)		

<<Study>> First author, publication year, specific study name if available are presented. Updated: Updated data that were published later were available.

<<Phase>> NS: not specified.

<<Primary outcome>> OS: overall survival. PFS: progression-free survival. QOL: quality of life. RR: response rate. DCR: disease control rate. TTP: time to progression. AE: adverse event. G3/4PFS: PFS without grade 3/4 AE. G4PFS: PFS without grade 4 AE. Non-inf: Primary outcome was evaluated by non-inferiority analysis. NS: not specified. ¶: data for OS was not obtainable.

<<Pathology>> NSCLC: non-small cell lung cancer. NSq: non-squamous carcinoma. Ad: adenocarcinoma. #: The study was originally designed for NSCLC. However, we extracted data only for NSq or Ad because regimen included Pemetrexed or Bevacizumab.

<<Arm>> 3>> 2 (excluded), 4>> 2 (excluded): The original study evaluated three/four arms. However, only two arms were included for our analysis because one/two arm(s) evaluated regimen(s) out of our concern.

3>>2 (combined): The original study evaluated three arms. However, we combined two arms with different dose of Bevacizumab.

<<Stage>> Rec: recurrent.

<<Performance status>> ECOG: Eastern Cooperative Oncology Group performance status. KPS: Karnofsky Performance Status

<<Regimens>> CDDP: Cisplatin. CBDCA: Carboplatin. CDGP: Nedaplatin. DTX: Docetaxel. PTX: Paclitaxel. VNR: Vinorelbine. GEM: Gemcitabine. CPT-11: Irinotecan. PEM: Pemetrexed. S1: Tegafur gimeracil oteracil. d: day. po: oral administration. bid: twice daily.

<<Patients>> Numbers of patients randomized for evaluated arms.

<<Median Age>> When median age (years) is not available, average age (years) is presented instead.

### Characteristics of included studies

The included studies were reported from a variety of countries all over the world, most of which were European or East Asian nations, and the USA (Table 1). The articles were published during 2000–2015. Among 48 reports, 18 were phase II studies, 26 were phase III studies, 17 evaluated OS as primary endpoints, 21 included ECOG 0-1 cases, and other 21 included ECOG 0–2 cases. We regarded two studies as three-arm studies, two studies as four-arm studies, and the other 44 as two-arm studies. Therefore, we eventually evaluated 102 arms, of which 92 were platinum doublet and 10 were platinum triplet. Both CDDP + VNR and CBDCA + PTX were the most frequently used arms, and were evaluated in 15 studies, followed by CDDP + GEM evaluated in 14 studies. Median/average age of patients ranged from 51 to 75, of which 35 were in their 60 s. The number of randomized patients in each study ranged from 41 to 1252 with a median of 248, which totaled 16842 patients (Table 1).

According to the Cochrane Risk of Bias evaluation, all but one of the studies had at least one domain of high risk of bias (Supplementary Table 1). One study had high risk of selection bias due to randomization using an envelope method, 46 studies had high risk of performance bias due to a non-blinded study design, one study had high risk of attrition bias because 21% of the randomized patients did not receive the allocated medication, and two studies had high risk of reporting bias because the primary endpoint was not specified. Twenty-seven were marked to have a high risk of other bias for potential conflicts of interest because the studies were directly funded, authored, or advised by pharmaceutical companies (Supplementary Table 1).

### Efficacy analysis

**Figure 2:**
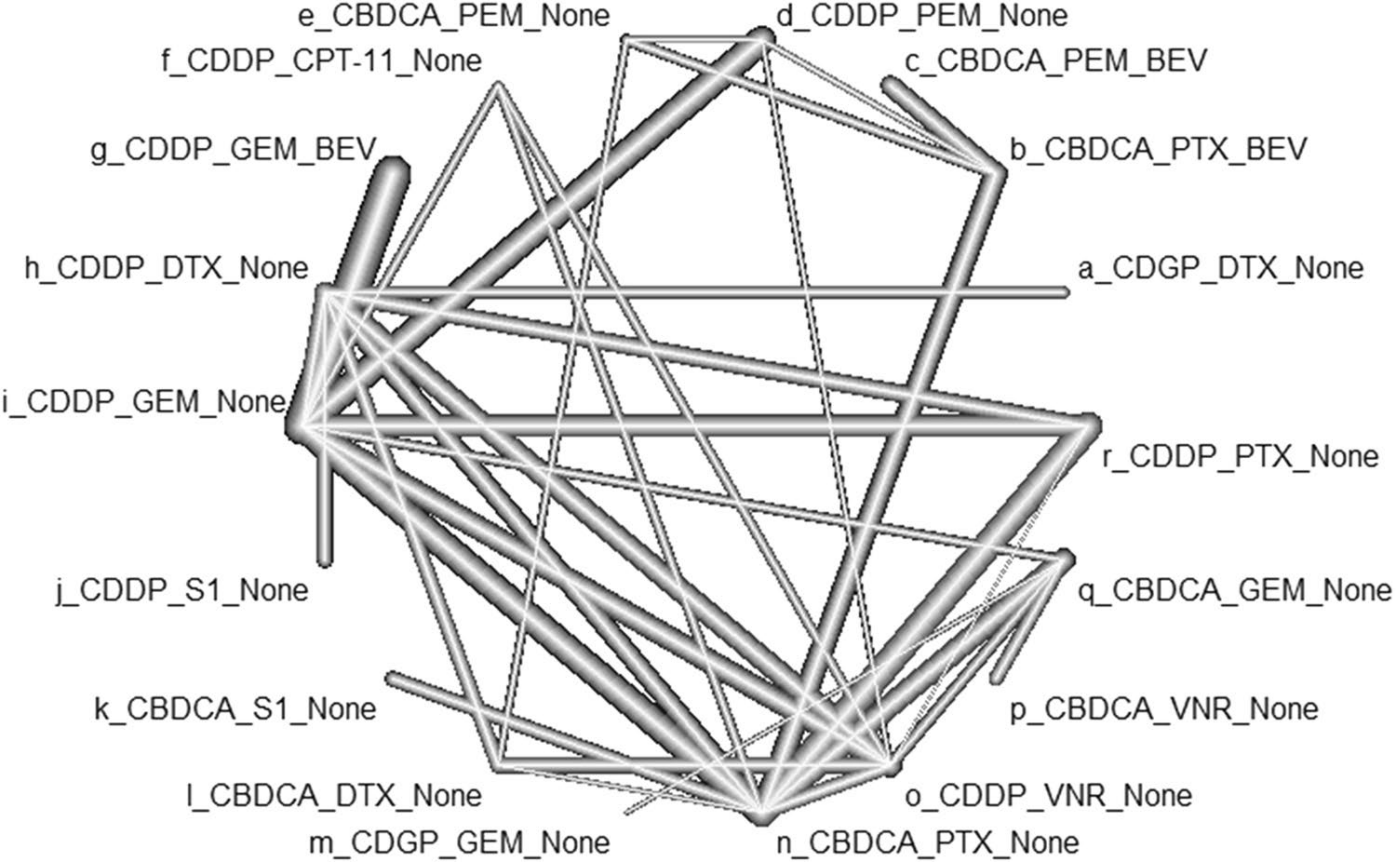
Network diagram for the primary endpoint, hazard ratio for overall survival. Q statistics and tests to assess consistency. Whole network (Q = 28.9, df = 34, P = 0.717). Within designs (Q = 11.8, df = 16, P = 0.760). Between design (Q = 17.1, df = 18, P = 0.516).

Data for HRos was obtainable in 45 studies with 16141 cases (Table 1, Fig. 2). HRs presented in 59 pairwise comparisons ranged from 0.68 to 1.22 with a median of 0.95. Q statics and a test for heterogeneity did not reveal inconsistency at any level: whole network level (Q = 28.9, df = 34, P = 0.717), within designs (Q = 11.8, df = 16, P = 0.760), and between design (Q = 17.1, df = 18, P = 0.516) (Fig. 2).

**Figure 3:**
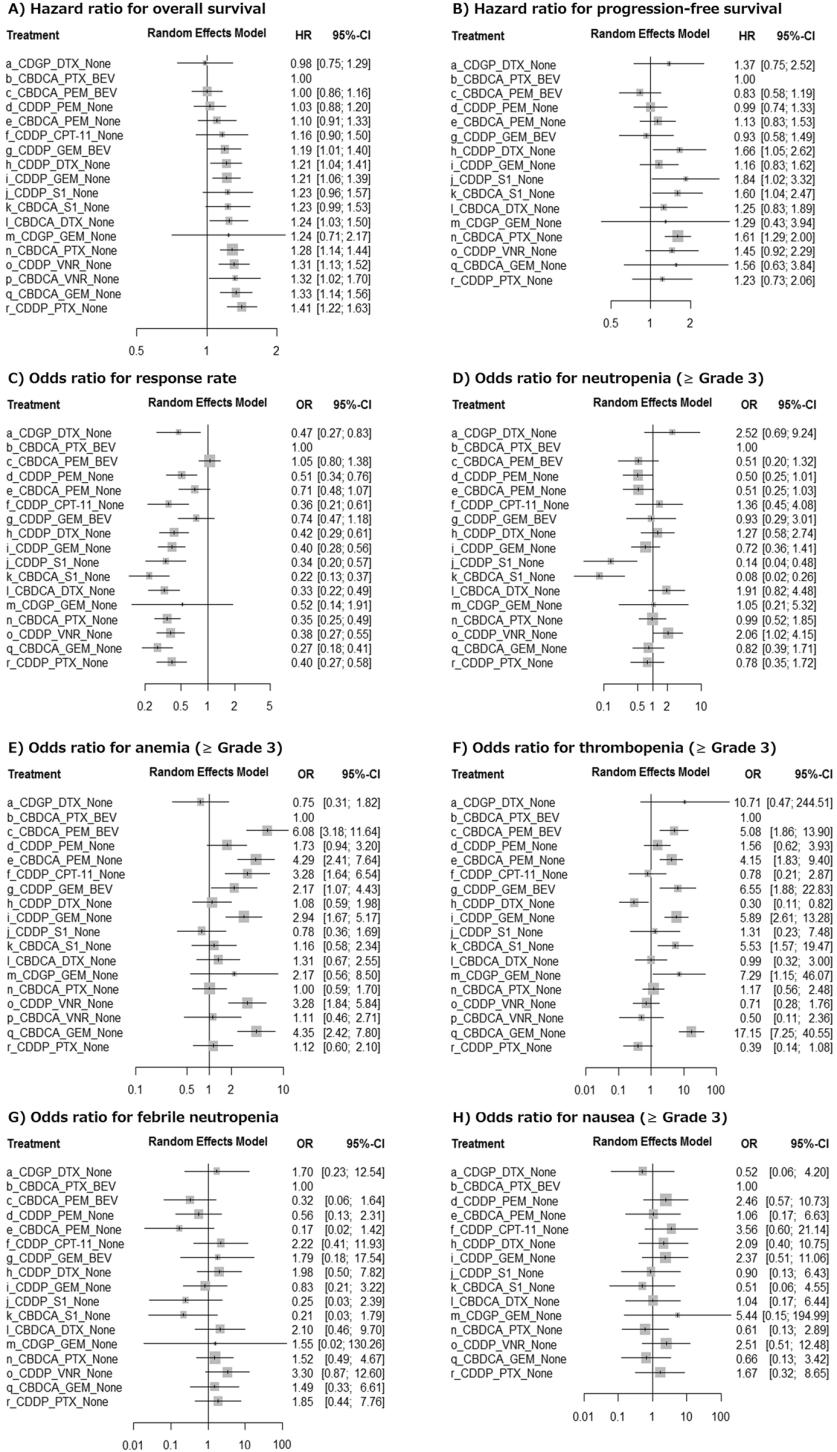
Forest plots for primary and secondary outcomes.

CDGP + DTX, which was evaluated in a phase III trial recruiting only squamous cancers, showed the best OS followed by CBDCA + PTX + BEV, CBDCA + PEM + BEV, CDDP + PEM, CBDCA + PEM, and CDDP + CPT-11 in this order (Fig. 3, Supplementary Table 2). HRos between any pair of these six regimens were not significant (P > 0.2, Supplementary Table 2). We selected CBDCA + PTX + BEV as the common comparator throughout this study because this regimen showed the best OS among the CDDP/CBDCA regimens. Using CBDCA + PTX + BEV as a common comparator, CDDP + S1 (HRos = 1.23, 95%CI: 0.96–1.57, P = 0.099), CBDCA + S1 (HRos = 1.23, 95%CI: 0.99–1.53, P = 0.062), and CDGP + GEM (HRos = 1.24, 95%CI: 0.71–2.17, P = 0.45) did not have significantly poorer HRos. Compared to CBDCA + PTX + BEV, the other regimens showed poorer survival assessed by HRos (P < 0.05 for all, Fig. 3, Supplementary Table 2).

Sensitivity analyses for HRos using the fixed model and using data only from phase III trials generally replicated the results (Supplementary Figure 1). A forest plot of HRos using CDGP + DTX and CDDP + CPT-11 as a common comparator is also shown to compare squamous NSCLC regimens (Supplementary Figure 1).

Three BEV regimens were high ranked for both PFS and RR. The lowest HRpfs was observed in CBDCA + PEM + BEV followed by CDDP + GEM + BEV, CDDP + PEM, and CBDCA + PTX + BEV in that order. The highest RR was shown by CBDCA + PEM + BEV followed by CBDCA + PTX + BEV, CDDP + GEM + BEV, and CBDCA + PEM. Notably, the CDGP + DTX arm had the best OS despite PFS and OR with lower ranks (Fig. 3).

### Safety analysis

Neutropenia was most frequently observed for CDGP + DTX and CDDP + VNR, while S1 regimens were associated with significantly less neutropenia (P < 0.01 for both). Patients treated by PEM and GEM regimens such as CBDCA + PEM + BEV, CBDCA + PEM, CDDP + GEM + BEV, CDDP + GEM, and CBDCA + GEM had a significantly higher risk of both grade III anemia and thrombopenia when compared to CBDCA + PTX + BEV (P < 0.05 for all). Lack of statistical power due to low occurrence made it difficult to detect the difference in risk for febrile neutropenia and nausea (Fig. 3).

## Discussion

We carried out the first network meta-analysis to compare platinum doublet and triplet regimens for chemo-naive incurable NSCLC. Among the 18 platinum regimens, CDGP + DTX, CBDCA + PTX + BEV, CBDCA + PEM + BEV, CDDP + PEM, and CDDP + CPT-11 in this rank order had the best performance in the primary endpoint, OS. In addition, we evaluated PFS, RR, and adverse events of grade III or higher as the secondary endpoints. The main advantage of this study over published systematic reviews on chemotherapy for NSCLC that used the conventional head-to-head meta-analysis is that we could compare a variety of chemotherapy regimens simultaneously by applying the network method (Fig. 2). In addition, the low heterogeneity (Fig. 2), the consistent results from sensitivity analyses (Supplementary Figure 1), the sound methodology following updated meta-analysis guidelines^4,11^, and the sufficient statistical power supported by the sufficient number of included studies and patients (Table 1) ensured the validity of the results. Although the results from this research could not recommend the single best regimen for NSCLC, we believe the current study provides useful data for the daily practice and for future chemotherapy trials.

For the treatment of non-squamous NSCLC, CBDCA + PTX + BEV and CBDCA + PEM + BEV resulted in the best OS (Fig. 3). The main SAEs concerned were anemia and thrombopenia by CBDCA + PEM + BEV and alopecia by CBDCA + PTX + BEV. Although PEM and CDDP are known to cause severe nausea and appetite loss, thanks to the recent development of anti-emesis drugs, PEM and CDDP regimens are no longer associated with severe nausea (Fig. 3). Although PFS and RR were inferior to regimens above, CDDP + PEM is another excellent regimen showing almost equivalent OS to these BEV regimens. The adverse event profile of CDDP + PEM was also similar to that of CBDCA + PTX + BEV. In the last few decades, it has been believed that daily hydration is mandatory for CDDP administration to avoid nephrotoxicity. However, the recent development of Mg-containing short hydration enables the administration of the CDDP regimen in an outpatient setting without a large amount of hydration^21^. Another advantage of the CDDP + PEM regimen is being able to avoid the economically expensive BEV. Actually, these three regimens have been often selected as the first-choice regimens. CBDCA + PEM is another promising regimen despite frequent anemia and thrombopenia. This regimen showed good indications for the elderly especially those with deteriorated renal function^22^. CDDP + CPT11 has been one of the classical standard regimens since it was shown to be superior to CDDP + Vindesine for treatment of NSCLC^23^. HRos by CDDP + CPT-11 compared to CBDCA + PTX + BEV was 1.16 (95%CI 0.90–1.50). This does not show significance; however, this may suggest that CDDP + CPT-11 is related to poorer survival. In addition, frequent severe diarrhea and anemia made it questionable to regard this regimen as the first choice. Imprecise estimation for OS by CDGP + GEM made the survival benefits of this regimen inconclusive. Given the non-promising results from the phase II study, we do not anticipate a phase III trial for CDGP + GEM in a non-squamous population. The other regimens had significantly or marginally significantly high HRos compared to CBDCA + PTX + BEV. Therefore, these regimens cannot be first-choice treatment for non-squamous NSCLC.

Oncologists require effective regimens for squamous NSCLC, which is not amenable to PEM, BEV, and tyrosine kinase inhibitors. A recently published trial by Shukuya *et al*. was the first study that reported the potential therapeutic effect of CDGP regimen for squamous NSCLC^13^. Their study could not show the statistical significance for OS evaluation compared to the control CDDP + DTX arm. However, in our analysis, this regimen resulted in the best OS among all the regimens analyzed (Fig. 3, Supplementary Table 2). Although neutropenia and thrombopenia were relatively common using CDGP + DTX, the adverse event profile was acceptable and could be compensated by the prolonged OS. Due to the lack of statistical power to prove OS improvement by the CDGP + DTX regimen, many of the established CDDP/CBDCA regimens remain as possible first choice treatment for squamous NSCLC treatment; though CBDCA + PTX (HR 1.31, 95%CI 1.02–1.68, P = 0.034), CDDP + VNR (HR 1.33, 95%CI 1.04–1.71, P = 0.025), CBDCA + GEM (HR 1.36, 95%CI 1.04–1.77, P = 0.023), and CDDP + PTX (HR 1.44, 95%CI 1.11–1.86, P = 0.006) led to poor OS compared to CDGP + DTX. The treatment choice among CDDP/CBDCA regimens is predominantly based on the adverse event profile and administration root, because there was not a large difference of OS among them. We anticipate further trials of CDGP + DTX and other CDGP regimens to establish a reliable regimen for squamous cell lung cancer.

A few limitations should be mentioned. First, some are skeptical about combining data from direct and indirect comparisons. However, the consistency between study designs dispels such doubt. Second, despite the very large sample size in the analysis, we could not clearly reveal the single best regimen. Nonetheless, the rank order among some first-choice regimens is informative. Third, platinum doublet and triplet treatments are often selected as second-line regimens after the failure of the first-line treatment by epidermal growth factor receptor-tyrosine kinase inhibitors, anaplastic lymphoma kinase inhibitors, and immune check-point inhibitors. Our study does not directly provide data for second-line platinum regimens. Fourth, most of the evaluated original trials had a high risk of bias judged from the Cochrane tool. Unfortunately, in practical terms, it is very difficult to conduct a double-blinded trial without sponsorship from pharmaceutical companies and we believe that these factors do not largely flaw the credibility of our analysis.

In conclusion, we conducted a systematic review and network meta-analysis. Based on 16842 NSCLC patients constituting 48 RCTs, CBDCA + PTX + BEV, CBDCA + PEM + BEV, CDDP + PEM, CBDCA + PEM, and CDDP + CPT-11 seemed reasonable first-choice regimens for non-squamous NSCLC. Even though other platinum regimes are also recommended in the guidelines, the results from our analysis do not support regular use of these regimens. CDGP + DTX and some CDDP/CBDCA regimens seemed acceptable first-choice regimens for squamous NSCLC.

## Electronic supplementary material

Supplementary File
