# Supplementary material for: The best platinum regimens for chemo-naive incurable non-small cell lung cancer: network meta-analysis
Source: Sci Rep. 2017 Oct 13;7:13185. doi: 10.1038/s41598-017-13724-2 (PMC5640659; doi:10.1038/s41598-017-13724-2)

**The best platinum regimens for chemo-naive incurable non-small cell lung cancer: network meta-analysis.**

**<<Supplementary file>>**

**Authors**

Nobuyuki Horita, Akimichi Nagashima, Kentaro Nakashima, Yuji Shibata, Kentaro Ito, Atsushi Goto, Takeharu Yamanaka, Takeshi Kaneko.

**Supplementary Text 1. Search formulas.**

*MEDLINE*

1 Carcinoma, Non-Small-Cell Lung/

2 (nscls or nsclc).tw.

3 "non small cell".tw.

4 (lung or pulmonary).tw.

5 (cancer* or neoplasm* or carcinoma* or malignan* or tumor* or tumour*).tw.

6 3 and 4 and 5

7 or/1-2,6

8 (advance* or metasta* or recurren* or inoperab* or relapse* or stage?3 or "stage 3" or stage3 or stage?III or "stage III" or stageIII or stage?4 or "stage 4" or stage4 or stage?IV or "stage IV" or stageIV).tw.

9 (naive or untreated or chemonaive or chemo-naive or non-treated or nontreated or first-line or front-line or primary or initial).tw.

10 7 and 8 and 9

11 exp Organoplatinum Compounds/

12 ("organoplatinum compound*" or platin*).tw.

13 (Carboplatin* or CBDCA).tw.

14 Cisplatin/

15 (Cisplatin or Dichlorodiammineplatinum* or cis-Diamminedichloroplatinum or cis-Diamminedichloroplatinum?II or cis-Platinum or CDDP).tw.

16 (Nedaplatin or CDGP or CGDP-2 or CGDP-II).tw.

17 or/11-16

18 Antineoplastic Agents/

19 (Antineoplastic or Anticancer or Antitumor or Cancer or Chemotherap*).tw.

20 (drug* or agent*).tw.

21 (third-generation or 3G or 3rd).tw.

22 (18 or (19 and 20)) and 21

23 (Irinotecan or camptothecin-11 or CPT-11 or SN-38* or 7-ethyl-10-hydroxycamptothecin or 7-ethyl-10-hydroxy-camptothecin or TOP).tw.

24 (Vinorelbine or "5'-nor-anhydrovinblastine" or "C'-Norvincaleukoblastine" or "3',4'-didehydro-4'-deoxy-" or KW-2307 or VNR).tw.

25 (Gemcitabine or dFdCyd or "2',2'-difluorodeoxycytidine" or "2'-deoxy-2'-difluorocytidine" or "2',2'-DFDC" or "2',2'-difluoro-2'-deoxycytidine" or LY-188011 or GEM).tw.

26 exp Paclitaxel/

27 (Paclitaxel or TXL or PTX or PAC XYOTAX or CT-2103 or P-PTX or PG-TXL or nab-PTX).tw.

28 (Docetaxel or docetaxol or DOC or DTX or TXT).tw.

29 Pemetrexed/

30 (Pemetrexed or PEM or MTA or LY-231514 or LY231514).tw.

31 (Amrubicin or AMR).tw.

32 (tegafur-gimeracil-oteracil or S1 or S-1).tw.

33 or/22-32

34 17 and 33

35 10 and 34

36 randomized controlled trial.pt.

37 controlled clinical trial.pt.

38 randomi?ed.ab.

39 placebo.ab.

40 clinical trials as topic.sh.

41 randomly.ab.

42 trial.ti.

43 groups.ab.

44 or/36-43

45 35 and 44

46 exp animals/ not humans.sh.

47 45 not 46

48 limit 47 to english language

*EMBASE*

#1 'non small cell lung cancer'/exp AND [embase]/lim NOT [medline]/lim

#2 nscls:ab,ti OR nsclc:ab,ti AND [embase]/lim NOT [medline]/lim

#3 'non small cell':ab,ti AND [embase]/lim NOT [medline]/lim

#4 lung:ab,ti OR pulmonary:ab,ti AND [embase]/lim NOT [medline]/lim

#5 cancer*:ab,ti OR neoplasm*:ab,ti OR carcinoma*:ab,ti OR malignan*:ab,ti OR tumor*:ab,ti OR tumour*:ab,ti AND [embase]/lim NOT [medline]/lim

#6 #3 AND #4 AND #5

#7 #1 OR #2 OR #6

#8 advance*:ab,ti OR metasta*:ab,ti OR recurren*:ab,ti OR inoperab*:ab,ti OR relapse*:ab,ti OR 'stage 3':ab,ti OR stage3:ab,ti OR 'stage iii':ab,ti OR stageiii:ab,ti OR 'stage 4':ab,ti OR stage4:ab,ti OR 'stage iv':ab,ti OR stageiv:ab,ti AND [embase]/lim NOT [medline]/lim

#9 naive:ab,ti OR untreated:ab,ti OR chemonaive:ab,ti OR 'chemo naive':ab,ti OR 'non treated':ab,ti OR nontreated:ab,ti OR 'first line':ab,ti OR 'front line':ab,ti OR primary:ab,ti OR initial:ab,ti AND [embase]/lim NOT [medline]/lim

#10 #7 AND #8 AND #9

#11 'platinum complex'/de AND [embase]/lim NOT [medline]/lim

#12 organoplatinum:ab,ti OR platin*:ab,ti AND [embase]/lim NOT [medline]/lim

#13 'carboplatin'/de OR carboplatin*:ab,ti OR cbdca:ab,ti AND [embase]/lim NOT [medline]/lim

#14 'cisplatin'/de AND cisplatin:ab,ti OR dichlorodiammineplatinum*:ab,ti OR 'cis diamminedichloroplatinum':ab,ti OR 'cis platinum':ab,ti OR cddp:ab,ti AND [embase]/lim NOT [medline]/lim

#15 'nedaplatin'/de OR nedaplatin:ab,ti OR cdgp:ab,ti OR 'cgdp-2':ab,ti OR 'cgdp-ii':ab,ti AND [embase]/lim NOT [medline]/lim

#16 #11 OR #12 OR #13 OR #14 OR #15

#17 'antineoplastic agent'/exp AND [embase]/lim NOT [medline]/lim

#18 antineoplastic:ab,ti OR anticancer:ab,ti OR antitumor:ab,ti OR cancer:ab,ti OR chemotherap*:ab,ti AND [embase]/lim NOT [medline]/lim

#19 drug*:ab,ti OR agent*:ab,ti AND [embase]/lim NOT [medline]/lim

#20 'third generation':ab,ti OR 3g:ab,ti OR 3rd:ab,ti AND [embase]/lim NOT [medline]/lim

#21 #17 OR (#18 AND #19) AND #20

#22 'irinotecan'/de OR irinotecan:ab,ti OR 'camptothecin 11':ab,ti OR 'cpt 11':ab,ti OR 'sn 38*':ab,ti OR '7 ethyl 10 hydroxycamptothecin':ab,ti OR '7 ethyl 10 hydroxy camptothecin':ab,ti OR top:ab,ti AND [embase]/lim NOT [medline]/lim

#23 'navelbine'/de OR vinorelbine:ab,ti OR '5-nor-anhydrovinblastine':ab,ti OR 'c-norvincaleukoblastine':ab,ti OR '3,4-didehydro-4-deoxy':ab,ti OR 'kw 2307':ab,ti OR vnr:ab,ti AND [embase]/lim NOT [medline]/lim

#24 'gemcitabine'/de OR gemcitabine:ab,ti OR dfdcyd:ab,ti OR '2,2-difluorodeoxycytidine':ab,ti OR '2-deoxy-2-difluorocytidine':ab,ti OR '2,2-dfdc':ab,ti OR '2,2-difluoro-2-deoxycytidine':ab,ti OR 'ly-188011':ab,ti OR gem:ab,ti AND [embase]/lim NOT [medline]/lim

#25 'paclitaxel'/de OR paclitaxel:ab,ti OR txl:ab,ti OR ptx:ab,ti OR 'pac xyotax':ab,ti OR 'ct-2103':ab,ti OR 'p-ptx':ab,ti OR 'pg-txl':ab,ti OR 'nab-ptx':ab,ti AND [embase]/lim NOT [medline]/lim

#26 'docetaxel'/de OR docetaxel:ab,ti OR docetaxol:ab,ti OR doc:ab,ti OR dtx:ab,ti OR txt:ab,ti AND [embase]/lim NOT [medline]/lim

#27 'pemetrexed'/de OR pemetrexed:ab,ti OR pem:ab,ti OR mta:ab,ti OR 'ly 231514':ab,ti OR ly231514:ab,ti AND [embase]/lim NOT [medline]/lim

#28 'amrubicin'/de OR amrubicin:ab,ti OR amr:ab,ti OR ly231514:ab,ti AND [embase]/lim NOT [medline]/lim

#29 'gimeracil plus oteracil potassium plus tegafur'/de OR 'tegafur gimeracil oteracil':ab,ti OR s1:ab,ti OR 's 1':ab,ti OR amr:ab,ti OR ly231514:ab,ti AND [embase]/lim NOT [medline]/lim

#30 #21 OR #22 OR #23 OR #24 OR #25 OR #26 OR #27 OR #28 OR #29

#31 #16 AND #30

#32 #10 AND #31

#33 'crossover procedure':de OR 'double-blind procedure':de OR 'randomized controlled trial':de OR 'single-blind procedure':de OR random*:de,ab,ti OR factorial*:de,ab,ti OR crossover*:de,ab,ti OR (cross NEXT/1 over*):de,ab,ti OR placebo*:de,ab,ti OR (doubl* NEAR/1 blind*):de,ab,ti OR (singl* NEAR/1 blind*):de,ab,ti OR assign*:de,ab,ti OR allocat*:de,ab,ti OR volunteer*:de,ab,ti AND [embase]/lim NOT [medline]/lim

#34 #33 NOT ([animals]/lim NOT [humans]/lim)

#35 #32 AND #34

*Web of Science*

Indexes=SCI-EXPANDED, CPCI-S Timespan=All years

# 1 TS=(("non small cell" AND (lung OR pulmonary) AND (cancer* OR neoplasm* OR carcinoma* OR malignan* OR tumor* OR tumour*)) OR (nscls OR nsclc))

# 2 TS=(advance* OR metasta* OR recurren* OR inoperab* OR relapse* OR stage?3 OR "stage 3" OR stage3 OR stage?III OR "stage III" OR stageIII OR stage?4 OR "stage 4" OR stage4 OR stage?IV OR "stage IV" OR stageIV)

# 3 TS=(naive or untreated OR chemonaive OR chemo-naive OR non-treated OR nontreated OR first-line OR front-line OR primary OR initial)

# 4 #3 AND #2 AND #1

# 5 TS=("organoplatinum compound*" OR platin* OR Carboplatin* OR CBDCA OR Cisplatin OR Dichlorodiammineplatinum* OR cis-Diamminedichloroplatinum OR cis-Diamminedichloroplatinum?II OR cis-Platinum OR CDDP OR Nedaplatin or CDGP OR CGDP-2 or CGDP-II)

# 6 TS=((Antineoplastic OR Anticancer OR Antitumor OR Cancer OR Chemotherap*) AND (drug* OR agent*) AND (third-generation OR 3G OR 3rd))

# 7 TS=(Irinotecan OR camptothecin-11 OR CPT-11 OR SN-38* OR 7-ethyl-10-hydroxycamptothecin OR 7-ethyl-10-hydroxy-camptothecin OR TOP)

# 8 TS=(Vinorelbine OR "5'-nor-anhydrovinblastine" OR "C'-Norvincaleukoblastine" OR "3',4'-didehydro-4'-deoxy-" OR KW-2307 OR VNR)

# 9 TS=(Gemcitabine OR dFdCyd OR "2',2'-difluorodeoxycytidine" OR "2'-deoxy-2'-difluorocytidine" OR "2',2'-DFDC" OR "2',2'-difluoro-2'-deoxycytidine" OR LY-188011 OR GEM)

# 10 TS=(Paclitaxel OR TXL OR PTX OR "PAC XYOTAX" OR "CT-2103" OR "P-PTX" OR "PG-TXL" OR "nab-PTX")

# 11 TS=(Docetaxel OR docetaxo OR DOC OR DTX OR TXT)

# 12 TS=(Pemetrexed OR PEM OR MTA OR LY-231514 OR LY231514)

# 13 TS=(Amrubicin OR AMR)

# 14 TS=(tegafur-gimeracil-oteracil OR S1 OR S-1)

# 15 #14 OR #13 OR #12 OR #11 OR #10 OR #9 OR #8 OR #7 OR #6

# 16 #15 AND #5

# 17 #16 AND #4

# 18 TS= clinical trial* OR TS=research design OR TS=comparative stud* OR TS=evaluation stud* OR TS=controlled trial* OR TS=followup stud* OR TS=prospective stud* OR TS=random* OR TS=placebo* OR TS=(single blind*) OR TS=(double blind*)

# 19 #18 AND #17

*Cochrane CENTRAL*

#1 MeSH descriptor: [Carcinoma, Non-Small-Cell Lung] this term only

#2 (nscls or nsclc):ti,ab,kw in Trials

#3 "non small cell":ti,ab,kw in Trials

#4 (lung or pulmonary):ti,ab,kw in Trials

#5 (cancer* or neoplasm* or carcinoma* or malignan* or tumor* or tumour*):ti,ab,kw in Trials

#6 #3 and #4 and #5 in Trials

#7 #1 or #2 or #6 in Trials

#8 (advance* or metasta* or recurren* or inoperab* or relapse* or stage?3 or "stage 3" or stage3 or stage?III or "stage III" or stageIII or stage?4 or "stage 4" or stage4 or stage?IV or "stage IV" or stageIV):ti,ab,kw in Trials

#9 (naive or untreated or chemonaive or chemo-naive or non-treated or nontreated or first-line or front-line or primary or initial) (naive or untreated or chemonaive or chemo-naive or non-treated or nontreated or first-line or front-line or primary or initial):ti,ab,kw in Trials

#10 #7 and #8 and #9 in Trials

#11 MeSH descriptor: [Organoplatinum Compounds] explode all trees

#12 ("organoplatinum compound*" or platin*):ti,ab,kw in Trials

#13 (Carboplatin* or CBDCA):ti,ab,kw in Trials

#14 MeSH descriptor: [Cisplatin] this term only

#15 (Cisplatin or Dichlorodiammineplatinum* or cis-Diamminedichloroplatinum or cis-Diamminedichloroplatinum?II or cis-Platinum or CDDP):ti,ab,kw in Trials

#16 (Nedaplatin or CDGP or CGDP-2 or CGDP-II):ti,ab,kw in Trials

#17 #11 or #12 or #13 or #14 or #15 or #16 in Trials

#18 MeSH descriptor: [Antineoplastic Agents] this term only

#19 (Antineoplastic or Anticancer or Antitumor or Cancer or Chemotherap*):ti,ab,kw in Trials

#20 (drug* or agent*):ti,ab,kw in Trials

#21 (third-generation or 3G or 3rd):ti,ab,kw in Trials

#22 (#18 or (#19 and #20)) and #21 in Trials

#23 (Irinotecan or camptothecin-11 or CPT-11 or SN-38* or 7-ethyl-10-hydroxycamptothecin or 7-ethyl-10-hydroxy-camptothecin or TOP):ti,ab,kw in Trials

#24 (Vinorelbine or "5'-nor-anhydrovinblastine" or "C'-Norvincaleukoblastine" or "3',4'-didehydro-4'-deoxy-" or KW-2307 or VNR):ti,ab,kw in Trials

#25 (Gemcitabine or dFdCyd or "2',2'-difluorodeoxycytidine" or "2'-deoxy-2'-difluorocytidine" or "2',2'-DFDC" or "2',2'-difluoro-2'-deoxycytidine" or LY-188011 or GEM):ti,ab,kw in Trials

#26 MeSH descriptor: [Paclitaxel] explode all trees

#27 (Paclitaxel or TXL or PTX or PAC XYOTAX or CT-2103 or P-PTX or PG-TXL or nab-PTX):ti,ab,kw in Trials

#28 (Docetaxel or docetaxol or DOC or DTX or TXT):ti,ab,kw in Trials

#29 MeSH descriptor: [Pemetrexed] this term only

#30 (Pemetrexed or PEM or MTA or LY-231514 or LY231514):ti,ab,kw in Trials

#31 (Amrubicin or AMR):ti,ab,kw in Trials

#32 (tegafur-gimeracil-oteracil or S1 or S-1):ti,ab,kw in Trials

#33 #22 or #23 or #24 or #25 or #26 or #27 or #28 or #29 or #30 or #31 or #32 in Trials

#34 #17 and #33 in Trials

#35 #10 and #34 in Trials

**Supplementary Text 2. R commands.**

library(netmeta)

NMA_data<-read.table("NSCLC_netmeta.csv",header=T,sep=",")

NMA_data

NMA_netmeta<-netmeta(TE, seTE, treat1, treat2, studlab, data=NMA_data, sm="HR", comb.random=TRUE, ref="") # if necessary, change HR to OR

NMA_netmeta

print(NMA_netmeta, digit=2)

netgraph(NMA_netmeta, seq=c("a_CDGP_DTX_None", "b_CBDCA_PTX_BEV", "c_CBDCA_PEM_BEV", "d_CDDP_PEM_None", "e_CBDCA_PEM_None", "f_CDDP_CPT-11_None", "g_CDDP_GEM_BEV", "h_CDDP_DTX_None", "i_CDDP_GEM_None", "j_CDDP_S1_None", "k_CBDCA_S1_None", "l_CBDCA_DTX_None", "m_CDGP_GEM_None", "n_CBDCA_PTX_None", "o_CDDP_VNR_None", "p_CBDCA_VNR_None", "q_CBDCA_GEM_None", "r_CDDP_PTX_None"), alpha.transparency = 0, dim="2d")

print(netrank(NMA_netmeta,small.values="bad")) forest(NMA_netmeta, ref="f_CDDP_CPT-11_None", pooled="random")

decomp.design(NMA_netmeta)

**Supplementary Text 3. List of included studies.**

1. Bennouna JH, Libor;Krzakowski, Maciej;Kollmeier, Jens;Gervais, Radj;Dansin, Eric;Serke, Monika;Favaretto, Adolfo;Szczesna, Aleksandra;Cobo, Manuel;Ciuffreda, Libero;Jassem, Jacek;Nicolini, Mario;Ramlau, Rodryg;Amoroso, Domenico;Melotti, Barbara;Almodovar, Teresa;Riggi, Marcello;Caux, Noel-Raphael;Vaissiere, Nathalie;Tan, Eng-Huat**.** Oral vinorelbine plus cisplatin as first-line chemotherapy in nonsquamous non-small-cell lung cancer: final results of an International randomized phase II study (NAVotrial 01). Clinical lung cancer. 2014;15(4):258-65.

2. Biesma BW, A. N. M.;Vincent, A.;Dalesio, O.;Smit, H. J. M.;Stigt, J. A.;Smit, E. F.;van Felius, C. L.;van Putten, J. W. G.;Slaets, J. P. J.;Groen, H. J. M.;Dutch Chest Physician Study, Group**.** Quality of life, geriatric assessment and survival in elderly patients with non-small-cell lung cancer treated with carboplatin-gemcitabine or carboplatin-paclitaxel: NVALT-3 a phase III study. Annals of oncology : official journal of the European Society for Medical Oncology. 2011;22(7):1520-7.

3. Chang JW-CT, Thomas Chang-Yao;Yang, Cheng-Ta;Lin, Meng-Chih;Cheung, Yun-Chung;Liaw, Chung-Chi;Chen, Chih-Hung**.** A randomized study of gemcitabine plus cisplatin and vinorelbine plus cisplatin in patients with advanced non-small-cell lung cancer. Chang Gung medical journal. 2008;31(6):559-66.

4. Chen Y-MP, Reury-Perng;Shih, Jen-Fu;Tsai, Chun-Ming;Whang-Peng, Jacqueline**.** A randomized phase II study of docetaxel or vinorelbine in combination with cisplatin against inoperable, chemo-naive non-small-cell lung cancer in Taiwan. Lung cancer (Amsterdam, Netherlands). 2007;56(3):363-9.

5. Chen YMP, R. P.;Shih, J. F.;Lee, Y. C.;Lee, C. S.;Tsai, C. M.;Whang-Peng, J. A randomised phase II study of weekly paclitaxel or vinorelbine in combination with cisplatin against inoperable non-small-cell lung cancer previously untreated. British journal of cancer. 2004;90(2):359-65.

6. Chen Y-MP, Reury-Perng;Tsai, Chun-Ming;Whang-Peng, Jacqueline**.** A Phase II randomized study of paclitaxel plus carboplatin or cisplatin against chemo-naive inoperable non-small cell lung cancer in the elderly. Journal of thoracic oncology : official publication of the International Association for the Study of Lung Cancer. 2006;1(2):141-5.

7. Comella P, Frasci G, Panza N, Manzione L, De Cataldis G, Cioffi R, et al. Randomized trial comparing cisplatin, gemcitabine, and vinorelbine with either cisplatin and gemcitabine or cisplatin and vinorelbine in advanced non-small-cell lung cancer: interim analysis of a phase III trial of the Southern Italy Cooperative Oncology Group. J Clin Oncol. 2000;18(7):1451-7.

8. Douillard JYG, R.;Dabouis, G.;Le Groumellec, A.;D'Arlhac, M.;Spaeth, D.;Coudert, B.;Caillaud, D.;Monnier, A.;Clary, C.;Maury, B.;Mornet, M.;Riviere, A.;Clouet, P.;Couteau, C. Sequential two-line strategy for stage IV non-small-cell lung cancer: docetaxel-cisplatin versus vinorelbine-cisplatin followed by cross-over to single-agent docetaxel or vinorelbine at progression: final results of a randomised phase II study. Annals of oncology : official journal of the European Society for Medical Oncology. 2005;16(1):81-9.

9. Edelman MJ, Clark JI, Chansky K, Albain K, Bhoopalam N, Weiss GR, et al. Randomized phase II trial of sequential chemotherapy in advanced non-small cell lung cancer (SWOG 9806): carboplatin/gemcitabine followed by paclitaxel or cisplatin/vinorelbine followed by docetaxel. Clin Cancer Res. 2004;10(15):5022-6.

10. Fossella FP, Jose R.;von Pawel, Joachim;Pluzanska, Anna;Gorbounova, Vera;Kaukel, Eckhard;Mattson, Karin V.;Ramlau, Rodryg;Szczesna, Aleksandra;Fidias, Panagiotis;Millward, Michael;Belani, Chandra P. Randomized, multinational, phase III study of docetaxel plus platinum combinations versus vinorelbine plus cisplatin for advanced non-small-cell lung cancer: the TAX 326 study group. Journal of clinical oncology : official journal of the American Society of Clinical Oncology. 2003;21(16):3016-24.

11. Galetta DC, Saverio;Pisconti, Salvatore;Gebbia, Vittorio;Morabito, Alessandro;Borsellino, Nicola;Maiello, Evaristo;Febbraro, Antonio;Catino, Annamaria;Rizzo, Pietro;Montrone, Michele;Misino, Andrea;Logroscino, Antonio;Rizzi, Daniele;Di Maio, Massimo;Colucci, Giuseppe**.** Cisplatin/Pemetrexed Followed by Maintenance Pemetrexed Versus Carboplatin/Paclitaxel/Bevacizumab Followed by Maintenance Bevacizumab in Advanced Nonsquamous Lung Cancer: The GOIM (Gruppo Oncologico Italia Meridionale) ERACLE Phase III Randomized Trial. Clinical lung cancer. 2015;16(4):262-73.

12. Gebbia VL, Vito;Galetta, Domenico;Caruso M, Michele;Palomba, Giuseppe;Riccardi, Fernando;Borsellino, Nicolo;Carrozza, Francesco;Leo, Silvana;Ferrau, Francesco;Cinieri, Saverio;Mancuso, Gianfranco;Mancarella, Sergio;Colucci, Giuseppe**.** First-line cisplatin with docetaxel or vinorelbine in patients with advanced non-small-cell lung cancer: a quality of life directed phase II randomized trial of Gruppo Oncologico Italia Meridionale. Lung cancer (Amsterdam, Netherlands). 2010;69(2):218-24.

13. Gebbia VG, Domenico;Caruso, Michele;Verderame, Francesco;Pezzella, Giuseppe;Valdesi, Matteo;Borsellino, Nicolo;Pandolfo, Giuseppe;Durini, Ernesto;Rinaldi, Massimo;Loizzi, Michele;Gebbia, Nicola;Valenza, Roberto;Tirrito, Maria Lina;Varvara, Francesca;Colucci, Giuseppe;Gruppo Ocologico Italia, Meridionale**.** Gemcitabine and cisplatin versus vinorelbine and cisplatin versus ifosfamide+gemcitabine followed by vinorelbine and cisplatin versus vinorelbine and cisplatin followed by ifosfamide and gemcitabine in stage IIIB-IV non small cell lung carcinoma: a prospective randomized phase III trial of the Gruppo Oncologico Italia Meridionale. Lung cancer (Amsterdam, Netherlands). 2003;39(2):179-89.

14. Gronberg BHB, Roy M.;Flotten, Oystein;Amundsen, Tore;Brunsvig, Paal Fr;Hjelde, Harald H.;Kaasa, Stein;von Plessen, Christian;Stornes, Froydis;Tollali, Terje;Wammer, Finn;Aasebo, Ulf;Sundstrom, Stein**.** Phase III study by the Norwegian lung cancer study group: pemetrexed plus carboplatin compared with gemcitabine plus carboplatin as first-line chemotherapy in advanced non-small-cell lung cancer. Journal of clinical oncology : official journal of the American Society of Clinical Oncology. 2009;27(19):3217-24.

15. Helbekkmo NS, S. H.;Aasebo, U.;Brunsvig, P. Fr;von Plessen, C.;Hjelde, H. H.;Garpestad, O. K.;Bailey, A.;Bremnes, R. M.;Norwegian Lung Cancer Study, Group**.** Vinorelbine/carboplatin vs gemcitabine/carboplatin in advanced NSCLC shows similar efficacy, but different impact of toxicity. British journal of cancer. 2007;97(3):283-9.

16. Johnson DHF, Louis;Novotny, William F.;Herbst, Roy S.;Nemunaitis, John J.;Jablons, David M.;Langer, Corey J.;DeVore, Russell F., 3rd;Gaudreault, Jacques;Damico, Lisa A.;Holmgren, Eric;Kabbinavar, Fairooz**.** Randomized phase II trial comparing bevacizumab plus carboplatin and paclitaxel with carboplatin and paclitaxel alone in previously untreated locally advanced or metastatic non-small-cell lung cancer. Journal of clinical oncology : official journal of the American Society of Clinical Oncology. 2004;22(11):2184-91.

17. Kader YALC, Thierry;El-Nahas, Tamer;Sakr, Amr**.** Comparative study analyzing survival and safety of bevacizumab/carboplatin/paclitaxel and cisplatin/pemetrexed in chemotherapy-naive patients with advanced non-squamous bronchogenic carcinoma not harboring EGFR mutation. OncoTargets and therapy. 2013;6:803-9.

18. Kawahara MA, Shinji;Komuta, Kiyoshi;Yoshioka, Hiroshige;Kawasaki, Masayuki;Fujita, Yuka;Yonei, Toshiro;Ogushi, Fumitaka;Kubota, Kaoru;Nogami, Naoyuki;Tsuchiya, Michiko;Shibata, Kazuhiko;Tomizawa, Yoshio;Minato, Koichi;Fukuoka, Kazuya;Asami, Kazuhiro;Yamanaka, Takeharu;Japan Multinational Trial, Organization**.** Carboplatin plus either docetaxel or paclitaxel for Japanese patients with advanced non-small cell lung cancer. Anticancer research. 2013;33(10):4631-7.

19. Khodadad KK, Adnan;Esfahani-Monfared, Zahra;Karimi, Shirin;Seifi, Sharare**.** Comparing docetaxel plus Cisplatin with Paclitaxel plus Carboplatin in chemotherapy-naive patients with advanced non-small-cell lung cancer: a single institute study. Iranian journal of pharmaceutical research : IJPR. 2014;13(2):575-81.

20. Kubota KS, H.;Katakami, N.;Nishio, M.;Inoue, A.;Okamoto, H.;Isobe, H.;Kunitoh, H.;Takiguchi, Y.;Kobayashi, K.;Nakamura, Y.;Ohmatsu, H.;Sugawara, S.;Minato, K.;Fukuda, M.;Yokoyama, A.;Takeuchi, M.;Michimae, H.;Gemma, A.;Kudoh, S.;Tokyo Cooperative Oncology, Group**.** A randomized phase III trial of oral S-1 plus cisplatin versus docetaxel plus cisplatin in Japanese patients with advanced non-small-cell lung cancer: TCOG0701 CATS trial. Annals of oncology : official journal of the European Society for Medical Oncology / ESMO. 2015;26(7):1401-8.

21. Langer CL, Sigui;Schiller, Joan;Tester, William;Rapoport, Bernardo L.;Johnson, David H.;Eastern Cooperative Oncology, Group**.** Randomized phase II trial of paclitaxel plus carboplatin or gemcitabine plus cisplatin in Eastern Cooperative Oncology Group performance status 2 non-small-cell lung cancer patients: ECOG 1599. Journal of clinical oncology : official journal of the American Society of Clinical Oncology. 2007;25(4):418-23.

22. Martoni A, Marino A, Sperandi F, Giaquinta S, Di Fabio F, Melotti B, et al. Multicentre randomised phase III study comparing the same dose and schedule of cisplatin plus the same schedule of vinorelbine or gemcitabine in advanced non-small cell lung cancer. Eur J Cancer. 2005;41(1):81-92.

23. Mazzanti PM, Cristian;Rocchi, Marco B. L.;Mattioli, Rodolfo;Lippe, Paolo;Trivisonne, Raffaele;Buzzi, Franco;De Signoribus, Giorgio;Tuveri, Guido;Rossi, Giorgio;Di Lullo, Liberato;Sturba, Fabio;Morale, Donatella;Catanzani, Sergio;Pilone, Alberta;Bonsignori, Maurizio;Battelli, Tullio**.** Randomized, multicenter, phase II study of gemcitabine plus cisplatin versus gemcitabine plus carboplatin in patients with advanced non-small cell lung cancer. Lung cancer (Amsterdam, Netherlands). 2003;41(1):81-9.

24. Minami SK, Takashi;Shiroyama, Takayuki;Okafuji, Kohei;Hirashima, Tomonori;Uchida, Junji;Imamura, Fumio;Osaki, Tadashi;Nakatani, Takeshi;Ogata, Yoshitaka;Yamamoto, Suguru;Namba, Yoshinobu;Otsuka, Tomoyuki;Tachibana, Isao;Komuta, Kiyoshi;Kawase, Ichiro**.** Randomized Phase II trial of paclitaxel and carboplatin followed by gemcitabine switch-maintenance therapy versus gemcitabine and carboplatin followed by gemcitabine continuation-maintenance therapy in previously untreated advanced non-small cell lung cancer. BMC research notes. 2013;6:3.

25. Niho SK, Hideo;Nokihara, Hiroshi;Horai, Takeshi;Ichinose, Yukito;Hida, Toyoaki;Yamamoto, Nobuyuki;Kawahara, Masaaki;Shinkai, Tetsu;Nakagawa, Kazuhiko;Matsui, Kaoru;Negoro, Shunichi;Yokoyama, Akira;Kudoh, Shinzoh;Kiura, Katsuyuki;Mori, Kiyoshi;Okamoto, Hiroaki;Sakai, Hiroshi;Takeda, Koji;Yokota, Soichiro;Saijo, Nagahiro;Fukuoka, Masahiro;J. O. Study Group**.** Randomized phase II study of first-line carboplatin-paclitaxel with or without bevacizumab in Japanese patients with advanced non-squamous non-small-cell lung cancer. Lung cancer (Amsterdam, Netherlands). 2012;76(3):362-7.

26. Ohe YO, Y.;Kubota, K.;Tamura, T.;Nakagawa, K.;Negoro, S.;Nishiwaki, Y.;Saijo, N.;Ariyoshi, Y.;Fukuoka, M. Randomized phase III study of cisplatin plus irinotecan versus carboplatin plus paclitaxel, cisplatin plus gemcitabine, and cisplatin plus vinorelbine for advanced non-small-cell lung cancer: Four-Arm Cooperative Study in Japan. Annals of oncology : official journal of the European Society for Medical Oncology. 2007;18(2):317-23.

27. Okamoto IY, Hiroshige;Morita, Satoshi;Ando, Masahiko;Takeda, Koji;Seto, Takashi;Yamamoto, Nobuyuki;Saka, Hideo;Asami, Kazuhiro;Hirashima, Tomonori;Kudoh, Shinzoh;Satouchi, Miyako;Ikeda, Norihiko;Iwamoto, Yasuo;Sawa, Toshiyuki;Miyazaki, Masaki;Tamura, Kenji;Kurata, Takayasu;Fukuoka, Masahiro;Nakagawa, Kazuhiko**.** Phase III trial comparing oral S-1 plus carboplatin with paclitaxel plus carboplatin in chemotherapy-naive patients with advanced non-small-cell lung cancer: results of a west Japan oncology group study. Journal of clinical oncology : official journal of the American Society of Clinical Oncology. 2010;28(36):5240-6.

28. Patel JDS, Mark A.;Garon, Edward B.;Reynolds, Craig H.;Spigel, David R.;Olsen, Mark R.;Hermann, Robert C.;Jotte, Robert M.;Beck, Thaddeus;Richards, Donald A.;Guba, Susan C.;Liu, Jingyi;Frimodt-Moller, Bente;John, William J.;Obasaju, Coleman K.;Pennella, Eduardo J.;Bonomi, Philip;Govindan, Ramaswamy**.** PointBreak: a randomized phase III study of pemetrexed plus carboplatin and bevacizumab followed by maintenance pemetrexed and bevacizumab versus paclitaxel plus carboplatin and bevacizumab followed by maintenance bevacizumab in patients with stage IIIB or IV nonsquamous non-small-cell lung cancer. Journal of clinical oncology : official journal of the American Society of Clinical Oncology. 2013;31(34):4349-57.

29. Reck MvP, Joachim;Zatloukal, Petr;Ramlau, Rodryg;Gorbounova, Vera;Hirsh, Vera;Leighl, Natasha;Mezger, Jorg;Archer, Venice;Moore, Nicola;Manegold, Christian**.** Phase III trial of cisplatin plus gemcitabine with either placebo or bevacizumab as first-line therapy for nonsquamous non-small-cell lung cancer: AVAil. Journal of clinical oncology : official journal of the American Society of Clinical Oncology. 2009;27(8):1227-34.

30. Rodrigues-Pereira JK, Joo-Hang;Magallanes, Manuel;Lee, Dae Ho;Wang, Jie;Ganju, Vinod;Martinez-Barrera, Luis;Barraclough, Helen;van Kooten, Maximiliano;Orlando, Mauro**.** A randomized phase 3 trial comparing pemetrexed/carboplatin and docetaxel/carboplatin as first-line treatment for advanced, nonsquamous non-small cell lung cancer. Journal of thoracic oncology : official publication of the International Association for the Study of Lung Cancer. 2011;6(11):1907-14.

31. Rosell RG, U.;Betticher, D. C.;Keppler, U.;Macha, H. N.;Pirker, R.;Berthet, P.;Breau, J. L.;Lianes, P.;Nicholson, M.;Ardizzoni, A.;Chemaissani, A.;Bogaerts, J.;Gallant, G. Phase III randomised trial comparing paclitaxel/carboplatin with paclitaxel/cisplatin in patients with advanced non-small-cell lung cancer: a cooperative multinational trial. Annals of oncology : official journal of the European Society for Medical Oncology. 2002;13(10):1539-49.

32. Sandler AG, Robert;Perry, Michael C.;Brahmer, Julie;Schiller, Joan H.;Dowlati, Afshin;Lilenbaum, Rogerio;Johnson, David H. Paclitaxel-carboplatin alone or with bevacizumab for non-small-cell lung cancer. The New England journal of medicine. 2006;355(24):2542-50.

33. Scagliotti GVP, Purvish;von Pawel, Joachim;Biesma, Bonne;Vansteenkiste, Johan;Manegold, Christian;Serwatowski, Piotr;Gatzemeier, Ulrich;Digumarti, Raghunadharao;Zukin, Mauro;Lee, Jin S.;Mellemgaard, Anders;Park, Keunchil;Patil, Shehkar;Rolski, Janusz;Goksel, Tuncay;de Marinis, Filippo;Simms, Lorinda;Sugarman, Katherine P.;Gandara, David**.** Phase III study comparing cisplatin plus gemcitabine with cisplatin plus pemetrexed in chemotherapy-naive patients with advanced-stage non-small-cell lung cancer. Journal of clinical oncology : official journal of the American Society of Clinical Oncology. 2008;26(21):3543-51.

34. Scagliotti GVDM, F.;Rinaldi, M.;Crino, L.;Gridelli, C.;Ricci, S.;Matano, E.;Boni, C.;Marangolo, M.;Failla, G.;Altavilla, G.;Adamo, V.;Ceribelli, A.;Clerici, M.;Di Costanzo, F.;Frontini, L.;Tonato, M.;Italian Lung Cancer, Project**.** Phase III randomized trial comparing three platinum-based doublets in advanced non-small-cell lung cancer. Journal of clinical oncology : official journal of the American Society of Clinical Oncology. 2002;20(21):4285-91.

35. Schiller JH, Harrington D, Belani CP, Langer C, Sandler A, Krook J, et al. Comparison of four chemotherapy regimens for advanced non-small-cell lung cancer. N Engl J Med. 2002;346(2):92-8.

36. Schuette WHWG, Andreas;Sebastian, Martin;Andreas, Stefan;Muller, Thomas;Schneller, Folker;Guetz, Sylvia;Eschbach, Corinna;Bohnet, Sabine;Leschinger, Monika I.;Reck, Martin**.** A randomized phase II study of pemetrexed in combination with cisplatin or carboplatin as first-line therapy for patients with locally advanced or metastatic non-small-cell lung cancer. Clinical lung cancer. 2013;14(3):215-23.

37. Shukuya TY, Takeharu;Seto, Takashi;Daga, Haruko;Goto, Koichi;Saka, Hideo;Sugawara, Shunichi;Takahashi, Toshiaki;Yokota, Soichiro;Kaneda, Hiroyasu;Kawaguchi, Tomoya;Nagase, Seisuke;Oguri, Tetsuya;Iwamoto, Yasuo;Nishimura, Takashi;Hattori, Yoshihiro;Nakagawa, Kazuhiko;Nakanishi, Yoichi;Yamamoto, Nobuyuki;West Japan Oncology, Group**.** Nedaplatin plus docetaxel versus cisplatin plus docetaxel for advanced or relapsed squamous cell carcinoma of the lung (WJOG5208L): a randomised, open-label, phase 3 trial. The Lancet. Oncology. 2015;16(16):1630-8.

38. Smit EFvM, Jan P. A. M.;Lianes, Pilar;Debruyne, Channa;Legrand, Catherine;Schramel, Franz;Smit, Hans;Gaafar, Rabab;Biesma, Bonne;Manegold, Chris;Neymark, Niels;Giaccone, Giuseppe;European Organization for, Research;Treatment of Cancer Lung Cancer, Group**.** Three-arm randomized study of two cisplatin-based regimens and paclitaxel plus gemcitabine in advanced non-small-cell lung cancer: a phase III trial of the European Organization for Research and Treatment of Cancer Lung Cancer Group--EORTC 08975. Journal of clinical oncology : official journal of the American Society of Clinical Oncology. 2003;21(21):3909-17.

39. Sun JA, Js;Jung, Sh;Sun, J;Ha, Sy;Han, J;Park, K;Ahn, Mj**.** Pemetrexed Plus Cisplatin Versus Gemcitabine Plus Cisplatin According to Thymidylate Synthase Expression in Nonsquamous Non-Small-Cell Lung Cancer: A Biomarker-Stratified Randomized Phase II Trial. Journal of clinical oncology : official journal of the American Society of Clinical Oncology; 2015:2450-6.

40. Tan EHR, J.;Grodzki, T.;Schneider, C. P.;Gatzemeier, U.;Zatloukal, P.;Aitini, E.;Carteni, G.;Riska, H.;Tsai, Y. H.;Abratt, R. Global Lung Oncology Branch trial 3 (GLOB3): final results of a randomised multinational phase III study alternating oral and i.v. vinorelbine plus cisplatin versus docetaxel plus cisplatin as first-line treatment of advanced non-small-cell lung cancer. Annals of oncology : official journal of the European Society for Medical Oncology. 2009;20(7):1249-56.

41. Thomas PR, G;Gouva, S;Fournel, P;Léna, H;Caer, H;Perol, M;Berard, H;Bombaron, P;Vergnenegre, A;Kleisbauer, Jp**.** Randomized multicentric phase II study of carboplatin/gemcitabine and cisplatin/vinorelbine in advanced non-small cell lung cancer GFPC 99-01 study (Groupe français de pneumo-cancérologie). Lung cancer (Amsterdam, Netherlands); 2006:105-14.

42. Treat JAG, R.;Socinski, M. A.;Edelman, M. J.;Catalano, R. B.;Marinucci, D. M.;Ansari, R.;Gillenwater, H. H.;Rowland, K. M.;Comis, R. L.;Obasaju, C. K.;Belani, C. P.;Alpha Oncology Research, Network**.** A randomized, phase III multicenter trial of gemcitabine in combination with carboplatin or paclitaxel versus paclitaxel plus carboplatin in patients with advanced or metastatic non-small-cell lung cancer. Annals of oncology : official journal of the European Society for Medical Oncology. 2010;21(3):540-7.

43. Wu Y-LL, Shun;Cheng, Ying;Zhou, Caicun;Wang, Mengzhao;Qin, Shukui;Lu, You;Zhang, Yang;Zhu, Yunzhong;Song, Xiangqun;Wang, Xin;Barraclough, Helen;Zhang, Xiaoqing;Chi, Haidong;Orlando, Mauro**.** Efficacy and safety of pemetrexed/cisplatin versus gemcitabine/cisplatin as first-line treatment in Chinese patients with advanced nonsquamous non-small cell lung cancer. Lung cancer (Amsterdam, Netherlands). 2014;85(3):401-7.

44. Yang J-JZ, Qing;Liao, Ri-Qiang;Huang, Yi-Sheng;Xu, Chong-Rui;Wang, Zhen;Wang, Bin-Chao;Chen, Hua-Jun;Wu, Yi-Long**.** Nedaplatin/Gemcitabine Versus Carboplatin/Gemcitabine in Treatment of Advanced Non-small Cell Lung Cancer: A Randomized Clinical Trial. Chinese journal of cancer research = Chung-kuo yen cheng yen chiu. 2012;24(2):97-102.

45. Zatloukal PP, Lubos;Zemanova, Milada;Kolek, Vitezslav;Skrickova, Jana;Pesek, Milos;Fojtu, Hana;Grygarkova, Ivona;Sixtova, Dimka;Roubec, Jaromir;Horenkova, Eva;Havel, Libor;Prusa, Petr;Novakova, Leona;Skacel, Tomas;Kuta, Milan**.** Gemcitabine plus cisplatin vs. gemcitabine plus carboplatin in stage IIIb and IV non-small cell lung cancer: a phase III randomized trial. Lung cancer (Amsterdam, Netherlands). 2003;41(3):321-31.

46. Zhang X, Lu J, Xu J, Li H, Wang J, Qin Y, et al. Pemetrexed plus platinum or gemcitabine plus platinum for advanced non-small cell lung cancer: final survival analysis from a multicentre randomized phase II trial in the East Asia region and a meta-analysis. Respirology. 2013;18(1):131-9.

47. Zhou CW, Yi-Long;Chen, Gongyan;Liu, Xiaoqing;Zhu, Yunzhong;Lu, Shun;Feng, Jifeng;He, Jianxing;Han, Baohui;Wang, Jie;Jiang, Guoliang;Hu, Chunhong;Zhang, Hao;Cheng, Gang;Song, Xiangqun;Lu, You;Pan, Hongming;Zheng, Wenjuan;Yin, Anny-Yue**.** BEYOND: A Randomized, Double-Blind, Placebo-Controlled, Multicenter, Phase III Study of First-Line Carboplatin/Paclitaxel Plus Bevacizumab or Placebo in Chinese Patients With Advanced or Recurrent Nonsquamous Non-Small-Cell Lung Cancer. Journal of clinical oncology : official journal of the American Society of Clinical Oncology. 2015;33(19):2197-204.

48. Zinner RGO, Coleman K.;Spigel, David R.;Weaver, Robert W.;Beck, J. Thaddeus;Waterhouse, David M.;Modiano, Manuel R.;Hrinczenko, Borys;Nikolinakos, Petros G.;Liu, Jingyi;Koustenis, Andrew G.;Winfree, Katherine B.;Melemed, Symantha A.;Guba, Susan C.;Ortuzar, Waldo I.;Desaiah, Durisala;Treat, Joseph A.;Govindan, Ramaswamy;Ross, Helen J. PRONOUNCE: randomized, open-label, phase III study of first-line pemetrexed + carboplatin followed by maintenance pemetrexed versus paclitaxel + carboplatin + bevacizumab followed by maintenance bevacizumab in patients ith advanced nonsquamous non-small-cell lung cancer. Journal of thoracic oncology : official publication of the International Association for the Study of Lung Cancer. 2015;10(1):134-42.

**Supplementary Table 1. The Cochrane Risk of Bias evaluation sheet.**

|  | Selection bias | Performance bias | Detection bias | Attrition bias | Reporting bias | Other bias |
| --- | --- | --- | --- | --- | --- | --- |
| Bennouna (2014) | Unclear | High | Low | Low | Low | High |
| Biesma (2011) | Unclear | High | Low | Low | Low | High |
| Chang (2008) | Unclear | High | Low | Low | Low | Low |
| Chen (2004) | Unclear | High | Low | Low | High | Low |
| Chen (2006) | Low | High | Low | Low | Low | Low |
| Chen (2007) | Unclear | High | Low | Low | Low | Low |
| Comella (2000) | Low | High | Low | Low | Low | Low |
| Douillard (2005) | Unclear | High | Low | Low | Low | High |
| Edelman (2004) | Low | High | Low | Low | Low | Low |
| Fossella (2003) | Low | High | Low | Low | Low | High |
| Galetta (2015) | Unclear | High | Low | Low | Low | Low |
| Gebbia (2003) | Low | High | Low | Low | Low | Low |
| Gebbia (2010) | Unclear | High | Low | Low | Low | Low |
| Gronberg (2009) | Low | High | Low | Low | Low | High |
| Helbekkmo (2007) | Low | High | Low | Low | Low | Low |
| Johnson (2004) | Low | High | Low | Low | Low | High |
| Kader (2013) | High | High | Low | Low | Low | Low |
| Kawahara (2013) | Low | High | Low | Low | Low | Low |
| Khodadad (2014) | Unclear | High | Low | Low | Low | High |
| Kubota (2015) | Low | High | Low | Low | Low | High |
| Langer (2007) | Low | High | Low | Low | Low | Low |
| Martoni (2005) | Unclear | High | Low | Low | Low | Low |
| Mazzanti (2003) | Unclear | High | Low | Low | Low | High |
| Minami (2013) | Unclear | High | Low | Low | Low | Low |
| Niho (2012) | Low | High | Low | Low | Low | High |
| Ohe (2007) | Low | High | Low | Low | Low | High |
| Okamoto (2010) | Low | High | Low | Low | Low | High |
| Patel (2013) | Unclear | High | Low | Low | Low | High |
| Reck (2009) | Low | Low | Low | Low | Low | High |
| Rodrigues (2011) | Low | High | Low | Low | Low | High |
| Rosell (2002) | Low | High | Low | Low | Low | High |
| Sandler (2006) | Unclear | High | Low | Low | Low | Low |
| Scagliotti (2002) | Low | High | Low | Low | High | High |
| Scagliotti (2008) | Unclear | High | Low | Low | Low | High |
| Schiller (2002) | Unclear | High | Low | Low | Low | Low |
| Schuette (2013) | Low | High | Low | Low | Low | High |
| Shukuya (2015) | Low | High | Low | Low | Low | High |
| Smit (2003) | Low | High | Low | Low | Low | High |
| Sun (2015) | Unclear | High | Low | Low | Low | High |
| Tan (2009) | Low | High | Low | Low | Low | Low |
| Thomas (2006) | Unclear | High | Low | Low | Low | High |
| Treat (2010) | Unclear | High | Low | Low | Low | High |
| Wu (2014) | Unclear | High | Low | Low | Low | High |
| Yang (2012) | Unclear | High | Low | High | Low | Low |
| Zatloukal (2003) | Unclear | High | Low | Low | Low | Low |
| Zhang (2013) | Low | High | Low | Low | Low | High |
| Zhou (2015) | Low | Low | Low | Low | Low | Low |
| Zinner (2015) | Unclear | High | Low | Low | Low | High |

**Supplementary Table 2. League table of hazard ratio for overall survival.**

|  | CDGP+DTX | CBDCA +PTX +BEV | CBDCA +PEM +BEV | CDDP +PEM | CBDCA +PEM | CDDP +CPT-11 | CDDP +GEM +BEV | CDDP +DTX | CDDP +GEM | CDDP +S1 | CBDCA +S1 | CBDCA +DTX | CDGP +GEM | CBDCA +PTX | CDDP +VNR | CBDCA +VNR | CBDCA +GEM | CDDP +PTX |
| --- | --- | --- | --- | --- | --- | --- | --- | --- | --- | --- | --- | --- | --- | --- | --- | --- | --- | --- |
| CDGP+DTX | NA | 0.98 (0.75-1.29) 0.884 | 0.98 (0.72-1.34) 0.899 | 0.95 (0.73-1.25) 0.709 | 0.89 (0.65-1.21) 0.462 | 0.84 (0.61-1.18) 0.300 | 0.82 (0.63-1.07) 0.142 | 0.81 (0.65-1.01) 0.061 | 0.81 (0.63-1.04) 0.099 | 0.80 (0.60-1.07) 0.131 | 0.80 (0.58-1.09) 0.166 | 0.79 (0.60-1.03) 0.087 | 0.79 (0.43-1.44) 0.445 | 0.76 (0.59-0.98) 0.034 | 0.75 (0.59-0.96) 0.021 | 0.74 (0.53-1.04) 0.080 | 0.74 (0.56-0.96) 0.029 | 0.69 (0.54-0.9) 0.004 |
| CBDCA+PTX+BEV | 1.02 (0.78-1.34) 0.886 | NA | 1.00 (0.86-1.16) 1.000 | 0.97 (0.83-1.14) 0.707 | 0.91 (0.75-1.09) 0.323 | 0.86 (0.67-1.11) 0.242 | 0.84 (0.71-0.99) 0.040 | 0.83 (0.71-0.96) 0.015 | 0.82 (0.72-0.95) 0.005 | 0.82 (0.64-1.04) 0.109 | 0.81 (0.65-1.01) 0.061 | 0.80 (0.67-0.97) 0.018 | 0.81 (0.46-1.41) 0.461 | 0.78 (0.69-0.88) <0.001 | 0.77 (0.66-0.89) <0.001 | 0.76 (0.59-0.98) 0.034 | 0.75 (0.64-0.88) <0.001 | 0.71 (0.61-0.82) <0.001 |
| CBDCA+PEM+BEV | 1.02 (0.75-1.39) 0.900 | 1.00 (0.86-1.16) 1.00 | NA | 0.97 (0.78-1.21) 0.786 | 0.91 (0.71-1.15) 0.443 | 0.86 (0.64-1.16) 0.320 | 0.84 (0.67-1.05) 0.128 | 0.83 (0.67-1.02) 0.082 | 0.82 (0.67-1.01) 0.058 | 0.82 (0.61-1.09) 0.18 | 0.81 (0.62-1.06) 0.124 | 0.80 (0.63-1.02) 0.069 | 0.81 (0.45-1.44) 0.478 | 0.78 (0.64-0.94) 0.011 | 0.77 (0.62-0.94) 0.014 | 0.76 (0.57-1.02) 0.065 | 0.75 (0.6-0.93) 0.010 | 0.71 (0.57-0.87) <0.001 |
| CDDP+PEM | 1.05 (0.80-1.37) 0.722 | 1.03 (0.88-1.20) 0.709 | 1.03 (0.83-1.28) 0.789 | NA | 0.93 (0.76-1.14) 0.483 | 0.89 (0.69-1.14) 0.363 | 0.87 (0.76-0.99) 0.039 | 0.85 (0.74-0.98) 0.023 | 0.85 (0.76-0.94) 0.003 | 0.84 (0.66-1.07) 0.157 | 0.84 (0.67-1.05) 0.128 | 0.83 (0.69-1.00) 0.049 | 0.83 (0.48-1.46) 0.511 | 0.80 (0.70-0.91) <0.001 | 0.79 (0.69-0.90) <0.001 | 0.78 (0.61-1.01) 0.053 | 0.77 (0.66-0.91) <0.001 | 0.73 (0.63-0.84) <0.001 |
| CBDCA+PEM | 1.13 (0.83-1.53) 0.433 | 1.10 (0.91-1.33) 0.325 | 1.10 (0.87-1.40) 0.432 | 1.07 (0.87-1.31) 0.517 | NA | 0.95 (0.71-1.28) 0.733 | 0.93 (0.75-1.15) 0.506 | 0.91 (0.74-1.12) 0.372 | 0.91 (0.75-1.11) 0.346 | 0.90 (0.68-1.19) 0.460 | 0.90 (0.69-1.18) 0.441 | 0.89 (0.72-1.10) 0.281 | 0.89 (0.50-1.59) 0.693 | 0.86 (0.71-1.04) 0.121 | 0.84 (0.69-1.03) 0.088 | 0.84 (0.62-1.12) 0.248 | 0.83 (0.67-1.03) 0.089 | 0.78 (0.63-0.96) 0.021 |
| CDDP+CPT-11 | 1.18 (0.85-1.65) 0.328 | 1.16 (0.90-1.5) 0.255 | 1.16 (0.86-1.56) 0.329 | 1.13 (0.88-1.45) 0.337 | 1.05 (0.78-1.41) 0.747 | NA | 0.98 (0.76-1.25) 0.874 | 0.96 (0.75-1.22) 0.742 | 0.96 (0.76-1.20) 0.726 | 0.95 (0.69-1.29) 0.748 | 0.95 (0.70-1.27) 0.736 | 0.93 (0.72-1.22) 0.590 | 0.94 (0.52-1.70) 0.838 | 0.9 (0.72-1.14) 0.369 | 0.89 (0.70-1.12) 0.331 | 0.88 (0.64-1.21) 0.431 | 0.87 (0.68-1.12) 0.274 | 0.82 (0.64-1.05) 0.116 |
| CDDP+GEM+BEV | 1.21 (0.93-1.58) 0.159 | 1.19 (1.01-1.40) 0.037 | 1.19 (0.95-1.48) 0.124 | 1.16 (1.01-1.32) 0.030 | 1.08 (0.87-1.34) 0.485 | 1.03 (0.80-1.31) 0.814 | NA | 0.98 (0.85-1.13) 0.781 | 0.98 (0.90-1.07) 0.647 | 0.97 (0.77-1.23) 0.799 | 0.97 (0.77-1.21) 0.792 | 0.96 (0.80-1.15) 0.659 | 0.96 (0.55-1.68) 0.886 | 0.93 (0.82-1.05) 0.250 | 0.91 (0.80-1.04) 0.159 | 0.90 (0.70-1.16) 0.414 | 0.89 (0.77-1.04) 0.129 | 0.84 (0.73-0.97) 0.016 |
| CDDP+DTX | 1.23 (0.99-1.55) 0.070 | 1.21 (1.04-1.41) 0.014 | 1.21 (0.98-1.50) 0.079 | 1.18 (1.02-1.36) 0.024 | 1.1 (0.89-1.35) 0.370 | 1.04 (0.82-1.33) 0.751 | 1.02 (0.88-1.17) 0.785 | NA | 1.00 (0.89-1.11) 1.000 | 0.99 (0.82-1.20) 0.918 | 0.99 (0.79-1.23) 0.929 | 0.97 (0.84-1.13) 0.687 | 0.98 (0.56-1.71) 0.943 | 0.94 (0.84-1.05) 0.277 | 0.93 (0.83-1.03) 0.188 | 0.92 (0.72-1.17) 0.501 | 0.91 (0.79-1.05) 0.194 | 0.86 (0.76-0.97) 0.015 |
| CDDP+GEM | 1.24 (0.96-1.59) 0.095 | 1.21 (1.06-1.39) 0.006 | 1.21 (0.99-1.49) 0.068 | 1.18 (1.06-1.31) 0.002 | 1.10 (0.90-1.34) 0.348 | 1.05 (0.83-1.32) 0.680 | 1.02 (0.94-1.11) 0.641 | 1.00 (0.90-1.12) 1.000 | NA | 0.99 (0.79-1.24) 0.930 | 0.99 (0.80-1.22) 0.926 | 0.98 (0.83-1.15) 0.808 | 0.98 (0.56-1.70) 0.943 | 0.95 (0.86-1.04) 0.290 | 0.93 (0.84-1.02) 0.143 | 0.92 (0.73-1.16) 0.480 | 0.91 (0.80-1.04) 0.159 | 0.86 (0.77-0.96) 0.007 |
| CDDP+S1 | 1.25 (0.93-1.68) 0.139 | 1.23 (0.96-1.57) 0.099 | 1.23 (0.92-1.64) 0.160 | 1.19 (0.94-1.51) 0.150 | 1.11 (0.84-1.47) 0.465 | 1.06 (0.78-1.44) 0.709 | 1.03 (0.81-1.31) 0.810 | 1.01 (0.84-1.23) 0.919 | 1.01 (0.81-1.26) 0.930 | NA | 1.00 (0.75-1.33) 1.000 | 0.99 (0.77-1.26) 0.936 | 0.99 (0.55-1.78) 0.973 | 0.96 (0.77-1.19) 0.713 | 0.94 (0.75-1.17) 0.585 | 0.93 (0.68-1.27) 0.649 | 0.92 (0.72-1.17) 0.501 | 0.87 (0.69-1.09) 0.233 |
| CBDCA+S1 | 1.25 (0.92-1.71) 0.158 | 1.23 (0.99-1.53) 0.062 | 1.23 (0.94-1.60) 0.127 | 1.19 (0.95-1.50) 0.135 | 1.11 (0.85-1.46) 0.450 | 1.06 (0.79-1.42) 0.697 | 1.03 (0.82-1.29) 0.798 | 1.01 (0.82-1.26) 0.928 | 1.01 (0.82-1.24) 0.925 | 1.00 (0.75-1.34) 1.000 | NA | 0.99 (0.77-1.26) 0.936 | 0.99 (0.56-1.77) 0.973 | 0.96 (0.79-1.15) 0.67 | 0.94 (0.76-1.16) 0.566 | 0.93 (0.7-1.25) 0.624 | 0.92 (0.74-1.14) 0.449 | 0.87 (0.71-1.07) 0.183 |
| CBDCA+DTX | 1.27 (0.97-1.66) 0.081 | 1.24 (1.03-1.50) 0.025 | 1.24 (0.98-1.58) 0.077 | 1.21 (1.00-1.45) 0.044 | 1.13 (0.91-1.39) 0.258 | 1.07 (0.82-1.40) 0.620 | 1.04 (0.87-1.25) 0.671 | 1.03 (0.88-1.19) 0.701 | 1.02 (0.87-1.20) 0.809 | 1.01 (0.79-1.29) 0.937 | 1.01 (0.79-1.29) 0.937 | NA | 1.00 (0.57-1.77) 1.000 | 0.97 (0.83-1.13) 0.699 | 0.95 (0.82-1.10) 0.494 | 0.94 (0.72-1.23) 0.651 | 0.93 (0.78-1.12) 0.432 | 0.88 (0.74-1.04) 0.141 |
| CDGP+GEM | 1.26 (0.69-2.3) 0.452 | 1.24 (0.71-2.17) 0.450 | 1.24 (0.69-2.21) 0.469 | 1.20 (0.69-2.1) 0.521 | 1.12 (0.63-2.00) 0.701 | 1.07 (0.59-1.93) 0.823 | 1.04 (0.60-1.82) 0.890 | 1.02 (0.59-1.78) 0.944 | 1.02 (0.59-1.77) 0.944 | 1.01 (0.56-1.82) 0.974 | 1.01 (0.57-1.80) 0.973 | 1.00 (0.57-1.76) 1.000 | NA | 0.96 (0.56-1.67) 0.884 | 0.95 (0.55-1.65) 0.855 | 0.94 (0.53-1.66) 0.832 | 0.93 (0.54-1.59) 0.792 | 0.88 (0.5-1.53) 0.654 |
| CBDCA+PTX | 1.31 (1.02-1.68) 0.034 | 1.28 (1.14-1.44) <0.001 | 1.28 (1.06-1.55) 0.011 | 1.25 (1.10-1.42) <0.001 | 1.16 (0.96-1.41) 0.130 | 1.11 (0.88-1.39) 0.371 | 1.08 (0.95-1.22) 0.228 | 1.06 (0.95-1.19) 0.311 | 1.06 (0.97-1.16) 0.202 | 1.05 (0.84-1.31) 0.667 | 1.05 (0.87-1.26) 0.606 | 1.03 (0.88-1.21) 0.716 | 1.04 (0.6-1.79) 0.888 | NA | 0.98 (0.89-1.09) 0.696 | 0.97 (0.78-1.22) 0.790 | 0.96 (0.86-1.08) 0.482 | 0.91 (0.83-1) 0.047 |
| CDDP+VNR | 1.33 (1.04-1.71) 0.025 | 1.31 (1.13-1.52) <0.001 | 1.31 (1.06-1.61) 0.011 | 1.27 (1.11-1.45) <0.001 | 1.18 (0.97-1.45) 0.107 | 1.13 (0.89-1.42) 0.305 | 1.10 (0.96-1.25) 0.157 | 1.08 (0.97-1.20) 0.156 | 1.08 (0.98-1.19) 0.12 | 1.07 (0.86-1.33) 0.543 | 1.06 (0.86-1.32) 0.594 | 1.05 (0.91-1.22) 0.514 | 1.06 (0.61-1.83) 0.835 | 1.02 (0.92-1.13) 0.706 | NA | 0.99 (0.78-1.26) 0.935 | 0.98 (0.86-1.12) 0.764 | 0.93 (0.82-1.05) 0.250 |
| CBDCA+VNR | 1.34 (0.96-1.87) 0.085 | 1.32 (1.02-1.7) 0.033 | 1.32 (0.98-1.77) 0.066 | 1.28 (0.99-1.65) 0.058 | 1.19 (0.89-1.6) 0.245 | 1.14 (0.83-1.56) 0.416 | 1.11 (0.86-1.42) 0.415 | 1.09 (0.85-1.39) 0.492 | 1.09 (0.86-1.37) 0.468 | 1.07 (0.79-1.47) 0.669 | 1.07 (0.8-1.44) 0.652 | 1.06 (0.81-1.39) 0.672 | 1.06 (0.60-1.89) 0.842 | 1.03 (0.82-1.29) 0.798 | 1.01 (0.80-1.28) 0.934 | NA | 0.99 (0.81-1.20) 0.920 | 0.93 (0.73-1.19) 0.560 |
| CBDCA+GEM | 1.36 (1.04-1.77) 0.023 | 1.33 (1.14-1.56) <0.001 | 1.33 (1.07-1.65) 0.01 | 1.29 (1.1-1.51) 0.002 | 1.21 (0.97-1.50) 0.087 | 1.15 (0.89-1.47) 0.275 | 1.12 (0.96-1.30) 0.143 | 1.10 (0.95-1.27) 0.198 | 1.10 (0.97-1.24) 0.128 | 1.09 (0.85-1.38) 0.486 | 1.08 (0.87-1.35) 0.492 | 1.07 (0.89-1.29) 0.475 | 1.08 (0.63-1.84) 0.778 | 1.04 (0.93-1.16) 0.487 | 1.02 (0.89-1.16) 0.770 | 1.01 (0.83-1.23) 0.921 | NA | 0.94 (0.82-1.08) 0.378 |
| CDDP+PTX | 1.44 (1.11-1.86) 0.006 | 1.41 (1.22-1.63) <0.001 | 1.41 (1.15-1.74) <0.001 | 1.37 (1.19-1.58) <0.001 | 1.28 (1.04-1.58) 0.021 | 1.22 (0.95-1.55) 0.111 | 1.19 (1.03-1.36) 0.014 | 1.17 (1.03-1.32) 0.013 | 1.16 (1.05-1.29) 0.005 | 1.15 (0.92-1.45) 0.228 | 1.15 (0.93-1.42) 0.195 | 1.14 (0.96-1.35) 0.132 | 1.14 (0.65-1.99) 0.646 | 1.10 (1.00-1.21) 0.05 | 1.08 (0.96-1.22) 0.208 | 1.07 (0.84-1.36) 0.582 | 1.06 (0.92-1.22) 0.418 | NA |

**Supplementary Figure 1. Forest plots for sensitivity analyses.**


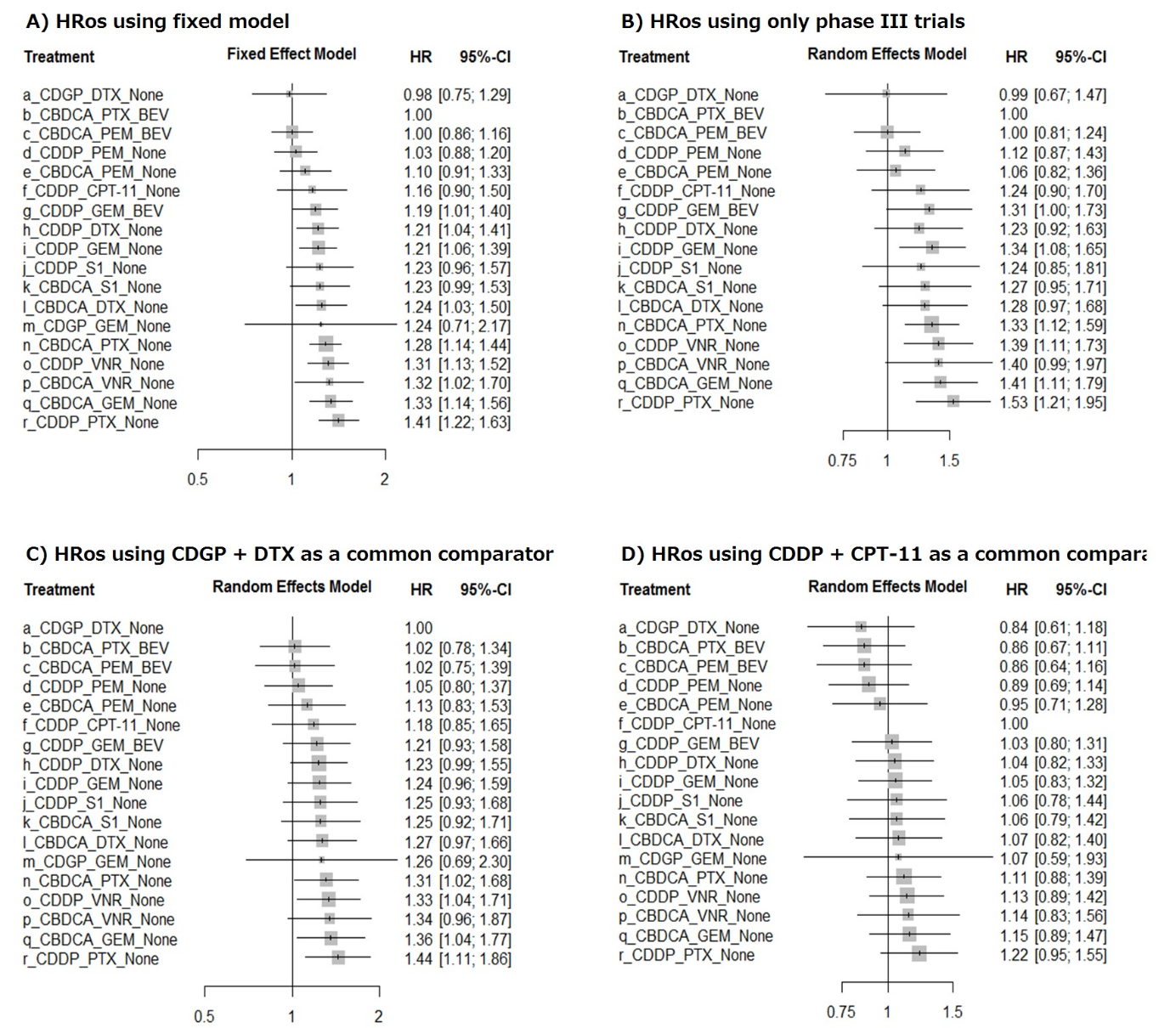

Supplement: Supplementary file 1 — Supplementary File [file 41598_2017_13724_MOESM1_ESM.doc]
